# Versatile Nano‐PROTAC‐Induced Epigenetic Reader Degradation for Efficient Lung Cancer Therapy

**DOI:** 10.1002/advs.202202039

**Published:** 2022-08-21

**Authors:** Huan‐Tian Zhang, Rui Peng, Sheng Chen, Ao Shen, Lixin Zhao, Wang Tang, Xiao‐He Wang, Zhen‐Yan Li, Zhen‐Gang Zha, Mengmeng Yi, Lingmin Zhang

**Affiliations:** ^1^ Department of Bone and Joint Surgery the First Affiliated Hospital Jinan University Guangzhou Guangdong 510630 P. R. China; ^2^ Key Laboratory of Molecular Target & Clinical Pharmacology and the State & NMPA Key Laboratory of Respiratory Disease School of Pharmaceutical Sciences & The Fifth Affiliated Hospital Guangzhou Medical University Guangzhou Guangdong 511436 P. R. China

**Keywords:** BRD4, epigenetic reader, PROTAC, tumor microenvironment, tumor‐associated macrophages

## Abstract

Recent evidence has indicated that overexpression of the epigenetic reader bromodomain‐containing protein 4 (BRD4) contributes to a poor prognosis of lung cancers, and the suppression of its expression promotes cell apoptosis and leads to tumor shrinkage. Proteolysis targeting chimera (PROTAC) has recently emerged as a promising therapeutic strategy with the capability to precisely degrade targeted proteins. Herein, a novel style of versatile nano‐PROTAC (CREATE (**CR** V‐LLC m **e** mbrane/DS‐PLG **A**/dB **ET** 6)) is developed, which is constructed by using a pH/GSH (glutathione)‐responsive polymer (disulfide bond‐linked poly(lactic‐*co*‐glycolic acid), DS‐PLGA) to load BRD4‐targeted PROTAC (dBET6), followed by the camouflage with engineered lung cancer cell membranes with dual targeting capability. Notably, CREATE remarkably confers simultaneous targeting ability to lung cancer cells and tumor‐associated macrophages (TAMs). The pH/GSH‐responsive design improves the release of dBET6 payload from nanoparticles to induce pronounced apoptosis of both cells, which synergistically inhibits tumor growth in both subcutaneous and orthotopic tumor‐bearing mouse model. Furthermore, the efficient tumor inhibition is due to the direct elimination of lung cancer cells and TAMs, which remodels the tumor microenvironment. Taken together, the results elucidate the construction of a versatile nano‐PROTAC enables to eliminate both lung cancer cells and TAMs, which opens a new avenue for efficient lung cancer therapy via PROTAC.

## Graphical abstract

A novel style of versatile nano‐proteolysis targeting chimera is constructed by the incorporation of the BRD4 degrader dBET6, which aims to target both lung cancer cells and M2 tumor‐associated macrophages (TAMs) which favors the tumor‐promoting microenvironment. Specific degradation of BRD4 leads to substantial cell apoptosis of both lung cancer cells and TAMs, thereby remodeling the tumor microenvironment, which in turn, renders tumor shrinkage. This work proposes a novel approach for specifically delivering the BRD4 degrader to regress cancers.

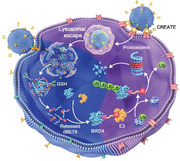

## 1 Introduction

Lung cancer is one of the most common malignant tumors with the highest morbidity and mortality.^[^ ^1^ ^]^ Recently, a number of epigenetic regulators have been identified as critical oncogenic drivers that can serve as therapeutic targets for lung cancer.^[^ ^2^ ^]^ However, these molecular targeted therapies are not applicable to cancers with mutations in the genes and/or less effective against patients with immune checkpoint‐negative tumors.^[^ ^3^ ^]^ Moreover, these therapies are mainly focused on the cancer cells, while the elimination of cancer cells alone does not remodel the tumor microenvironment (TME), which plays a fundamental role in promoting tumor growth and progression.^[^ ^1^, ^4^ ^]^

Tumor‐associated macrophages (TAMs) are a major type of infiltrating immune cells in lung cancers that act as a source of local and systemic cues to support the proliferation, survival, and migration of tumor cells.^[^ ^5^ ^]^ In addition, TAMs are polarized toward an M2‐like subtype in the TME during tumor progression. They can prevent the activation of a series of immune cells, such as dendritic cells, cytotoxic T lymphocytes, and natural killer cells, thus favoring tumor growth and survival via angiogenesis.^[^ ^6^ ^]^ Clinical observations have revealed that the accumulation of TAMs is correlated with poor prognosis in lung cancer. Given the important role of TAMs in orchestrating tumor progression, the strategies designed to diminish TAMs directly have become a promising approach for improving cancer therapy.^[^ ^7^ ^]^

Bromodomain‐containing proteins 4 (BRD4), a bromodomain and extra‐terminal (BET) protein, is well‐characterized epigenetic readers that recognize and bind to acetylated lysine residues and regulated many genes involved in diverse cellular functions including apoptosis.^[^ ^8^ ^]^ BRD4 is frequently found to be upregulated in lung cancers, and suppression of BRD4 leads to tumor shrinkage through inhibiting cell proliferation and/or causing cell apoptosis in a c‐Myc‐dependent and c‐Myc‐independent manner.^[^ ^9^ ^]^ Recently, the inhibition of BRD4 by JQ1 has been reported to block the proliferation of TAMs by reducing the expression and secretion of CSF1 by tumor cells.^[^ ^10^ ^]^ Thus, targeting the dysregulated epigenetic enzymes such as BRD4 has become an attractive approach for lung cancer therapy.

A series of BET inhibitors, including JQ1, BMS‐986158, OTX‐015, and GSK525762, have been developed and subjected to early clinical trials. However, their applications in the clinic are limited, especially for solid tumors, due to the relatively low specificity and various toxicities.^[^ ^11^ ^]^ Proteolysis targeting chimera (PROTAC) has emerged as a new and promising modality by utilizing an event‐driven mode of action, which draws inspiration from natural regulatory mechanisms and facilitates the elimination of native or degron‐tagged proteins from cells.^[^ ^12^ ^]^ Recent studies have demonstrated that BRD4 degraders, such as dBET6, enable to effectively induce apoptosis of human lung cancer cells with higher potencies than JQ1. However, dBET6 shows poor solubility and low bioavailability,^[^ ^13^ ^]^ which compromises their therapeutic effect on lung cancer.

**Scheme 1:**
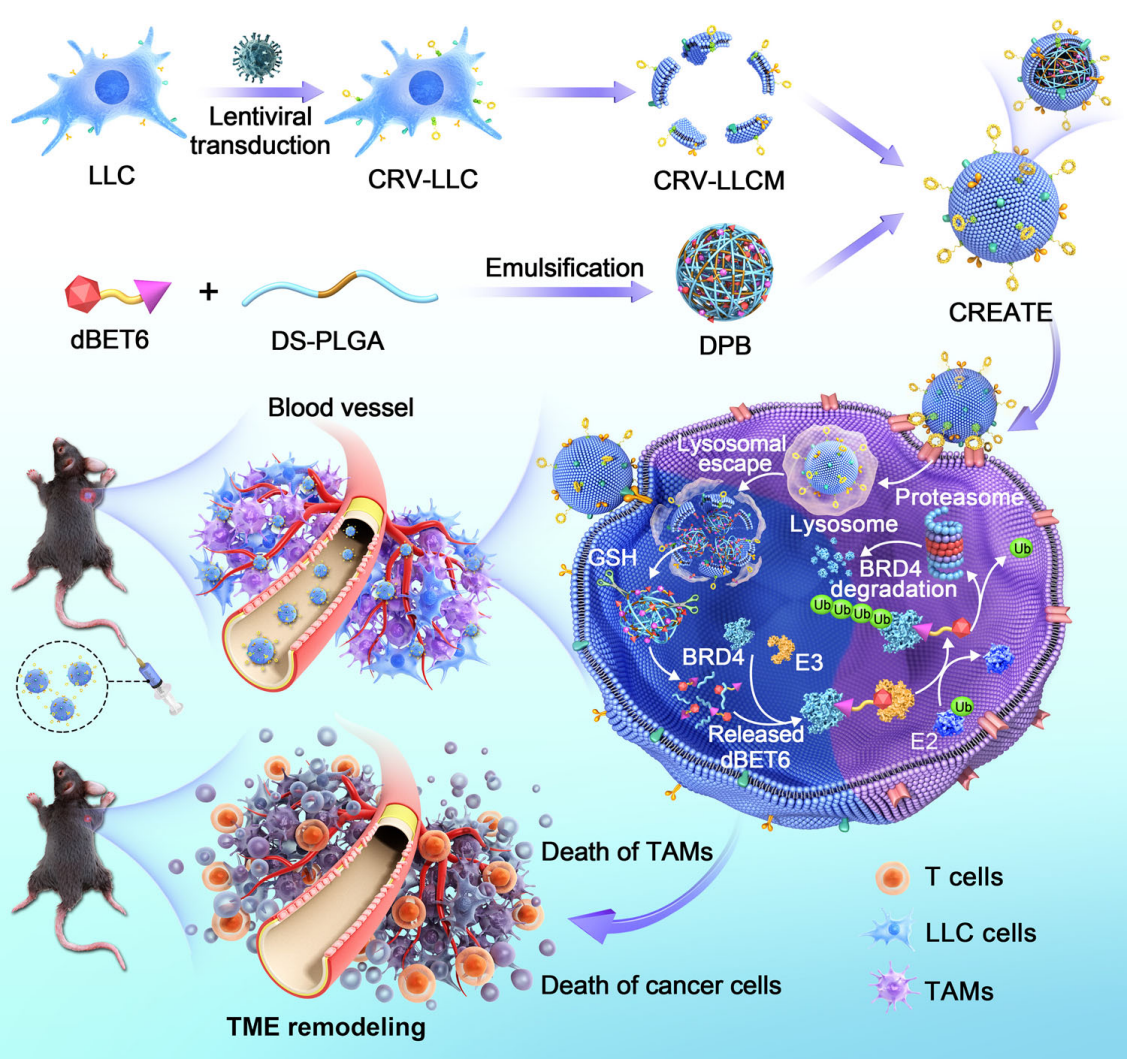
Schematic diagram of CRV‐LLCM‐coated nanoparticles formulation and the underlying therapeutic mechanisms.

In the present study, we developed a novel style of versatile nano‐PROTAC named CREATE to precisely degrade BRD4. The nanoparticles were constructed by the use of a pH/GSH‐responsive polymer (disulfide bond‐linked poly(lactic‐*co*‐glycolic acid), PLGA‐S‐S‐PLGA, hereafter refer as DS‐PLGA) to load dBET6, followed by the camouflage with CRV‐engineered Lewis lung carcinoma (LLC) cell membranes (CRV‐LLCM). We hypothesized that CREATE rendered simultaneous targeting ability to both cancer cells and TAMs due to the engineered membranes. As expected, the pH/GSH‐responsiveness significantly facilitated the release of dBET6 from the nanoparticles within the cells, which led to the efficient degradation of BRD4, inducing apoptosis of both cell lines and the inhibition of tumor growth both in vitro and in vivo (**Scheme** **1**). Our work provides solid evidence for developing novel strategies toward lung cancer therapy by using the versatile nano‐PROTAC.

## 2 Results

### 2.1 Preparation and Characterization of CREATE

Increasing evidence has shown that BRD4 plays an important role in lung cancer development. We consistently found that a high expression level of BRD4 was observed in lung adenocarcinoma, which predicted a poor prognosis in the Kaplan–Meier Plotter database (Figure S1, Supporting Information). Recently, we and others have demonstrated that inhibition of BRD4 by its inhibitor (JQ1) significantly suppresses cancer cell growth by triggering cell apoptosis and sensitizes lung cancer cells to radiotherapy.^[^ ^14^ ^]^ In this study, dBET6, a more potent BRD4 degrader developed from its predecessors dBET1 and JQ1,^[^ ^13^ ^]^ was selected to prepare nanoparticles aiming to increase therapeutic efficacy in lung cancer. PLGA‐based polymers exhibit good biocompatibility and high drug loading efficiency.^[^ ^15^ ^]^ and the modification endows PLGA with more specific functions. For example, DS‐PLGA with disulfide bond as linker was supposed to possess pH and glutathione (GSH) responsiveness, and thus were used for encapsulating dBET6. An optimized weight ratio of DS‐PLGA to dBET6 (DS‐PLGA/dBET6 = 15/1) was determined by assessing the loading and encapsulation efficiency (Figure S2, Supporting Information). A peptide denoted as CRV (a nine amino acid peptide CRVLRSGSC^[^ ^16^ ^]^) that selectively targeted to macrophages was overexpressed in LLC cells by the transduction with plasmid‐encoded CRV and Lamp2 (lysosome‐associated membrane protein‐2) fusion protein. Next, CRV‐LLCM was obtained to coat the nanoparticles aiming to target both lung cancer cells and TAMs.

**Figure 1:**
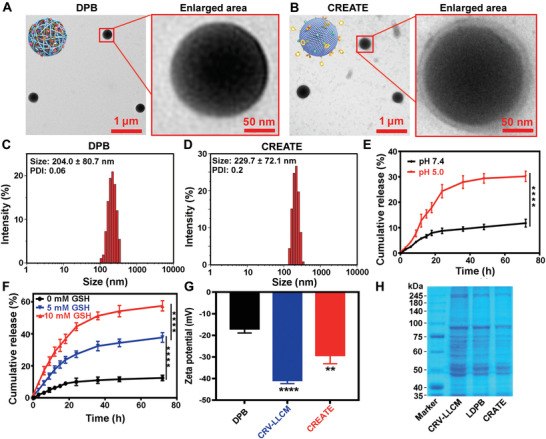
Characterizations of CRV‐LLCM‐based nanoparticles. A,B) TEM analysis of DPB and CREATE. C,D) DLS analysis of DPB and CREATE. E) Cumulative release of DPB in neutral (pH = 7.4) and acidic environment (pH = 5) (*n* = 3). F) Cumulative release of DPB in non‐GSH environment (GSH = 0 × 10^−3^ m) and GSH environment (GSH = 5 × 10^−3^ M and 10 × 10^−3^ m, respectively) (*n* = 3). G) Zeta potential of DPB, CRV‐LLCM, and CREATE (*n* = 3). H) SDS‐PAGE analysis of the protein composition of CRV‐LLCM, LDPB (LLC membrane/DS‐PLGA/dBET6), and CREATE, respectively. LDPB, LLCM/DS‐PLGA/dBET6; CREATE, CRV‐LLCM/DS‐PLGA/dBET6. Data are presented as mean ± SD. ** *p* < 0.01 and **** *p* < 0.0001.

By screening conditions for the formulation of CREATE, an optimized weight ratio of CRV‐LLCM to DPB (DS‐PLGA/dBET6) was found to be 3/1, as this ratio achieved a relatively small size and a good particle dispersity index (PDI) (Figure S3, Supporting Information). The transmission electron microscopy (TEM) analysis indicated that DPB was spherical with a diameter of ≈200 nm (**Figure** **1**A), while the DPB camouflaged by CRV‐LLCM (CREATE) showed a core–shell structure with a ≈10 nm thin layer (Figure 1B). This was confirmed by dynamic light scattering (DLS) analysis, which revealed that the hydrodynamic diameter of DPB was 204 ± 80.7 nm (Figure 1C), while CREATE increased to 229.71 ± 72.1 nm (Figure 1D). In addition, we examined the stability of nanoparticles and found that the size of CREATE remained stable for up to 9 d (Figure S4, Supporting Information). The in vitro release curve showed that dBET6 was released from DPB with ≈2‐fold faster rate at pH 5.0 than at pH 7.4 within 24 h (Figure 1E). Furthermore, TEM analysis indicated that the DS‐PLGA based nanoparticles were collapsed into irregular structure at pH 6.5 or 5.0 (Figure S5, Supporting Information). Similarly, the nanoparticles were also collapsed when these nanoparticles were exposed to high GSH concentrations. Significantly, the combination of low pH and high GSH concentration accelerated the degradation of DS‐PLGA based nanoparticles (Figure S5, Supporting Information). The lung cancer cells generally exhibit a reductive environment through fine‐tuning the GSH level ranging from 2 to 10 × 10^−3^ m in the cytosol.^[^ ^17^ ^]^ We then mimicked the environment by different concentration of GSH and found that GSH increased from 0 to 10 × 10^−3^ m triggered a gradual increase in the cumulative release rate (Figure 1F), confirming the GSH responsiveness of the prepared formulation. We also measured the average zeta potentials of DPB, CRV‐LLCM, and CREATE, which was corresponding to −17, −41, and −29 mV, respectively (Figure 1G). Sodium dodecyl sulfate‐polyacrylamide gel electrophoresis (SDS‐PAGE) confirmed the presence and enrichment of key surface antigens derived from CRV‐LLCM onto the nanoparticles (Figure 1H and Figure S6A, Supporting Information). Furthermore, we detected high level expression of Lamp2 proteins on CRV‐LLCM by immunoblotting, implying that CRV was also overexpressed for it was fused into Lamp2 (Figure S6B, Supporting Information). Overall, we have successfully established pH/GSH dual‐responsive BRD4 PROTAC nanoparticles, which may improve the therapeutic efficiency induced by dBET6.

### 2.2 CREATE Induces Lung Cancer Cells Apoptosis in 2D versus 3D Culture System

**Figure 2:**
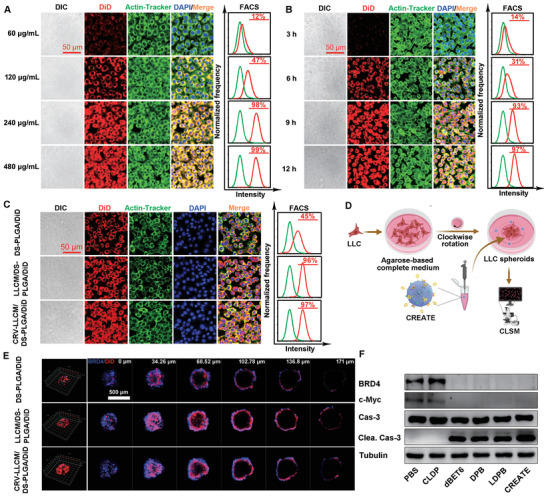
Biological effect induced by different formulations. A) Dose‐dependent cellular uptake of different formulations determined by CLSM (left panel) and FACS (right panel). LLC cells were treated with various concentrations of CRV‐LLCM/DS‐PLGA/DiD (CRV‐LLCM/DS‐PLGA/DiD was equivalent to 60, 120, 240, and 480 µg mL^−1^, respectively) for 9 h. B) Time‐dependent cellular uptake of different formulations determined by CLSM (left panel) and FACS (right panel). LLC cells were treated with CRV‐LLCM/DS‐PLGA/DiD (240 µg mL^−1^) for 3, 6, 9, and 12 h. C) Cellular uptake of DS‐PLGA/DiD, LLCM/DS‐PLGA/DiD, and CRV‐LLCM/DS‐PLGA/DiD. LLC cells were treated with the indicated nanoparticles (DS‐PLGA/DiD equivalent to 60 µg mL^−1^; LLCM/DS‐PLGA/DiD and CRV‐LLCM/DS‐PLGA/DiD equivalent to 240 µg mL^−1^) for 9 h. D) Schematic diagram of LLC spheroids formulation and treatment. E) Cellular uptake evaluation of DS‐PLGA/DiD, LLCM/DS‐PLGA/DiD, and CRV‐LLCM/DS‐PLGA/DiD in LLC spheroids. The 3D tumor spheroids were treated with the indicated formulations (DS‐PLGA/DiD equivalent to 60 µg mL^−1^; LLCM/DS‐PLGA/DiD and CRV‐LLCM/DS‐PLGA/DiD were equivalent to 240 µg mL^−1^) for 9 h. F) Immunoblotting analysis of BRD4, c‐Myc, and apoptotic genes in LLC cells. The cells treated with different formulations (PBS, CLDP (CRV‐LLC membrane/DS‐PLGA/NC), dBET6, DPB, LDPB, or CREATE, respectively) for 9 h, and incubated with the freshly prepared medium for another 15 h. Tubulin served as a loading control.

Next, we sought to evaluate the inhibition of cancer cells induced by CREATE. Because dBET6 did not have fluorescence properties, then the fluorescent dye 1,1′‐dioctadecyl‐3,3,3′,3′‐tetramethylindodicarbocyanine 4‐chlorobenzenesulfonate salt (DiD) was applied as an indicator to visualize the distribution of various formulations in LLC cells. As expected, the cellular uptake of CRV‐LLCM/DS‐PLGA/DiD occurred in a dose‐ and time‐dependent manner, ranging from 60 to 480 µg mL^−1^, 3 to 12 h, respectively. Notably, the optimal dose of CRV‐LLCM/DS‐PLGA/DiD (240 µg mL^−1^) and incubation time (9 h) were identified by confocal laser scanning microscopy (CLSM) and flow cytometry (FACS) analysis (**Figure** **2**A,B) and thus were selected for the following experiments. CLSM and FACS analysis indicated that the camouflage with LLCM or CRV‐LLCM enhanced the internalization of nanoparticles (Figure 2C). Similar results were observed in a 3D cell culture system using Z‐stack analysis (Figure 2D,E). In agreement with this, rapid BRD4 degradation was observed upon the internalization of dBET6, DPB, LDPB, and CREATE (Figure 2F). Moreover, we confirmed that depletion of BRD4 suppressed the expression of c‐Myc and induced apoptosis in lung cancer.^[^ ^18^ ^]^

**Figure 3:**
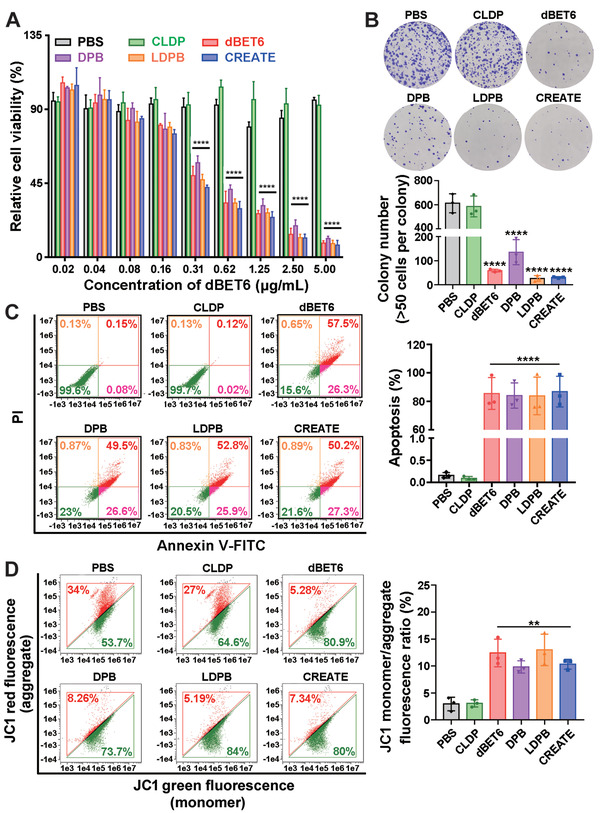
Inhibitory effects induced by different formulations in vitro. A) Cell viability of LLC cells. B) Colony formation assays of LLC cells treated with different formulations. LLC cells were treated with different formulations (PBS, CLDP, dBET6, DPB, LDPB, or CREATE, respectively) for 9 h, and incubated with the freshly prepared medium for another 7 d (*n* = 3). C) Annexin V‐FITC/propidium iodide (PI) apoptosis assay of LLC cells. D) Quantification of JC1 monomers/aggregates of LLC cells. To evaluate the cell viability and apoptosis, LLC cells were treated with different formulations (PBS, CLDP, dBET6, DPB, LDPB, or CREATE, respectively) for 9 h, and incubated with the freshly prepared medium for another 15 h (*n* = 3). Data are presented as mean ± SD. ** *p* < 0.01 and **** *p* < 0.0001.

Next, we investigated how dBET6‐containing formulations might affect cancer cell growth. Of significance, the dBET6‐containing formulations suppressed lung cancer cell growth, as evaluated by Cell Counting Kit‐8 (CCK‐8) and colony formation assays (**Figure** **3**A,B), implying that the BRD4 degradation effectively inhibited the cancer cell growth. Furthermore, the Live/Dead assay indicated that the dBET6‐containing formulations induced pronounced cell death as determined by the Live/Dead assay (Figure 3C and Figure S7, Supporting Information). JC1 staining also confirmed a decrease in mitochondrial membrane potential, a hallmark of the early stages of apoptosis (Figure 3D and Figure S8, Supporting Information). Interestingly, we found that the nano‐PROTAC constructed with DS‐PLGA led to a significantly higher apoptotic rate of the LLC cells and M2‐like macrophages, compared with the one with pH‐responsive but GSH‐nonresponsive materials (Figure S9, Supporting Information), indicating that the DS‐PLGA exhibits an excellent performance in controlled release of dBET6 thereby triggering more cell apoptosis. These results together suggest that the BRD4 degrader controls cell death mainly through a caspase‐3‐related apoptosis pathway.

### 2.3 Elimination of TAMs by dBET6‐Containing Formulations

**Figure 4:**
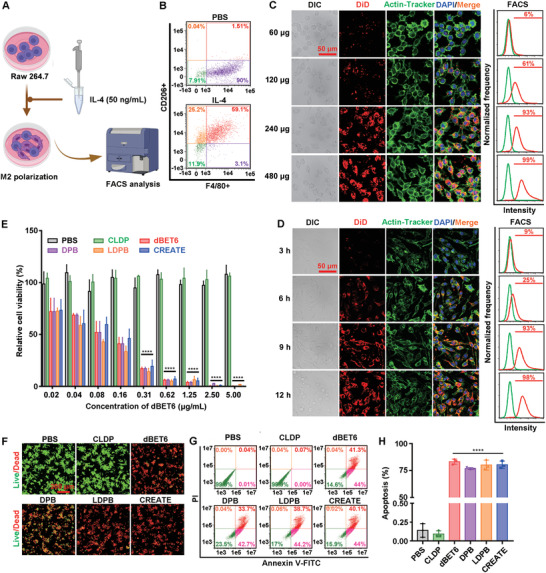
Inhibitory effects induced by different formulations on M2‐like macrophages in vitro. A) Schematic diagram of the induction and identification of M2‐like macrophages. B) FACS analysis of IL‐4‐induced (50 ng mL^−1^ for 48 h) M2‐like macrophages after incubation with anti‐F4/80 and anti‐CD206 antibodies. C) Dose‐dependent cellular uptake of nanoparticles in M2‐like macrophages assessed by CLSM (left panel) and FACS (right panel). M2‐like macrophages were treated with various concentrations of CRV‐LLCM/DS‐PLGA/DiD (CRV‐LLCM/DS‐PLGA/DiD equivalent to 60, 120, 240, and 480 µg mL^−1^, respectively) for 9 h. D) Time‐dependent cellular uptake of nanoparticles in M2‐like macrophages assessed by CLSM (left panel) and FACS (right panel). LLC cells were treated with CRV‐LLCM/DS‐PLGA/DiD (240 µg mL^−1^) for 3, 6, 9, and 12 h. E) Cell viability of M2‐like macrophages. F) Live/Dead analysis of M2‐like macrophages. G) Annexin V‐FITC/ propidium iodide (PI) apoptosis of M2‐like macrophages. H) Quantification of apoptotic rate (%) of M2‐like macrophages. To evaluate the cell viability, Live/Dead analysis, and apoptosis, M2‐like macrophages were treated with different formulations (PBS, CLDP, dBET6, DPB, LDPB, or CREATE, respectively) for 9 h, and incubated with the freshly prepared medium for another 15 h (*n* = 3). Data are presented as mean ± SD. **** *p* < 0.0001.

Increasing evidence has suggested that TAMs are polarized towards an M2‐like subtype and provide support for lung cancer growth and survival. Accordingly, direct elimination of TAMs has been proposed as a promising strategy for cancer therapy.^[^ ^7^ ^]^ Previous study have reported that BRD4 inhibition by JQ1 blocks the proliferation of TAMs.^[^ ^10^ ^]^ To this end, M2‐like macrophages were induced by treating the Raw 264.7 cells with IL‐4, and further validated by the F4/80^+^ and CD206^+^ staining by FACS (**Figure** **4**A,B). Next, the cellular uptake of CRV‐LLCM/DS‐PLGA/DiD by M2‐like macrophages was examined, and unsurprisingly, the M2‐like macrophages also showed a high level of cellular uptake (Figure 4C,D). CLSM and FACS analysis indicated that the camouflage with CRV‐LLCM mediated the most effective internalization by M2‐like macrophages (Figure S10, Supporting Information). Treatment of M2‐like cells with different concentrations of dBET6 and a panel of nanoparticles led to a significant reduction in the relative cell viability determined by CCK‐8 assay (Figure 4E). Live/Dead assay indicated that the dBET6‐containing formulations induced significant cell death of M2‐like cells (Figure 4F and Figure S11, Supporting Information). Furthermore, the dBET6‐containing formulations were found to induce significant cell apoptosis (Figure 4G,H), which was similar to the performance in LLC cells.

Accumulated evidence has suggested that colony‐stimulating factor 1 receptor (CSF1R), the receptor for colony stimulating factor 1, is highly expressed in TAMs and monocytes. By utilizing CSF1R, the CSF1/CSF1R signaling was reported to promote the proliferation and differentiation of TAMs.^[^ ^10^, ^19^ ^]^ To demonstrate the potential mechanism for the inhibition of M2‐like macrophages by the nano‐PROTAC, we firstly evaluated the changes in CSF1R mRNA level of the M2‐like macrophages derived from Raw 264.7 or bone marrow‐derived macrophages (BMDMs). Briefly, the M2‐like macrophages were treated with different concentrations of nano‐PROTAC (60, 120, or 240 µg mL^−1^) for 9 h, and incubated with the freshly prepared medium for another 15 h, followed by the detection of CSF1R mRNA levels by RT‐PCR analysis. Of note, a dose‐dependent decrease of the CSF1R mRNA expression was observed upon the treatment of the nano‐PROTAC in Raw 264.7‐induced M2‐like macrophages (Figure S12, Supporting Information). Similar results were obtained using BMDMs for M2‐like macrophage induction (Figure S12, Supporting Information). Expression of the M2 marker CD206 (also named MRC1) was concomitantly downregulated by the nano‐PROTAC in the cells (Figure S12, Supporting Information). CSF1R blockade was demonstrated to specifically inhibit the proliferation and survival of murine M2‐like macrophages.^[^ ^20^, ^21^ ^]^ In agreement with this, a dose‐dependent reduction of mitochondrial membrane potential (JC1 ratio quantification by flow cytometry) was observed when treated with different concentrations of CREATE (Figure S13, Supporting Information). These results together suggest that CREATE can trigger M2‐like macrophages to undergo cell apoptosis via suppressing the expression of CSF1R and MRC1 in a tumor‐free environment.

### 2.4 Effect of dBET6‐Containing Formulations on M2/LLC Spheroids

**Figure 5:**
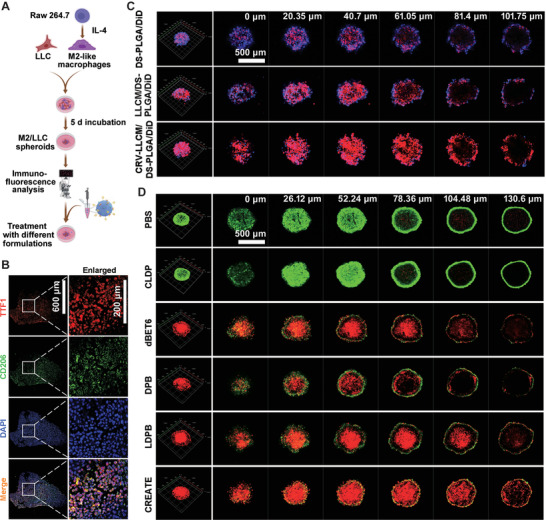
Biological effects induced by different formulations on M2/LLC spheroids. A) Schematic diagram of M2/LLC spheroids formulation and treatment. B) Verification of M2/LLC spheroids using antibodies against TTF1 and CD206. C) Cellular uptake of DS‐PLGA/DiD, LLCM/DS‐PLGA/DiD, or CRV‐LLCM/DS‐PLGA/DiD in M2/LLC spheroids. The M2/LLC spheroids were treated with the indicated formulations (DS‐PLGA/DiD equivalent to 60 µg mL^−1^; LLCM/DS‐PLGA/DiD, or CRV‐LLCM/DS‐PLGA/DiD equivalent to 240 µg mL^−1^) for 9 h. The M2/LLC spheroid was conducted with Z‐stack scanning with the distance between slices of 20.35 m. D) Live/Dead assay analyzing M2/LLC spheroids treated with different formulations (PBS, CLDP, dBET6, DPB, LDPB, or CREATE, respectively) for 9 h, and incubated with the freshly prepared medium for another 15 h (*n* = 3). The spheroids were conducted with Z‐stack scanning with the distance between slices of 26.12 μm.

Given the above‐mentioned findings of CREATE, we then evaluated its tumor suppressive effect in 3D tumor spheroid assay. We were the first to establish the TAMs‐lung cancer cell spheroids (M2/LLC spheroids) by incubating the M2‐like macrophages with LLC cells (**Figure** **5**A), followed by the verification using antibodies against TTF1 (the marker for LLC cells) and CD206 (the marker for TAMs) by immunofluorescence staining (Figure 5B). CLSM analysis indicated that CRV‐LLCM/DS‐PLGA/DiD led to a much higher infiltration in the 3D M2/LLC spheroids when compared with DS‐PLGA/DiD or LLCM/DS‐PLGA/DiD (Figure 5C). The penetration of CRV‐LLCM/DS‐PLGA/DiD provoked significant cell death within the spheroid, as evidenced by the Live/Dead assay (Figure 5D).

To further explore the endocytosis mechanism of CREATE by the LLC cells and M2‐like macrophages, a series of endocytosis inhibitors including chlorpromazine (CPZ, clathrin‐mediated endocytosis inhibitor), amiloride (AMI, micropinocytosis inhibitor), and genistein (GEN, caveolin‐mediated endocytosis inhibitor) was applied. Since temperature is a key factor that affects cellular uptake, we also determined the cellular uptake efficiency by placing cells at 4 °C individually. Of note, uptake of CREATE by the cells was markedly inhibited at 4 °C (Figures S14–S17, Supporting Information), implying that the endocytosis process was energy‐dependent. Furthermore, treatment of the LLC cells and M2‐like macrophages with GEN significantly reduced the cellular uptake of CREATE to ≈45% and ≈30%, whereas the addition of CPZ or AMI to the cells showed negligible effects (Figures S14–S17, Supporting Information), indicating that CREATE was internalized primarily through the caveolin‐dependent endocytosis, which was directly involved in the internalization of membrane components or extracellular ligands.^[^ ^22^, ^23^ ^]^ These results collectively suggest that the dual‐targeting membrane‐camouflaged nanoparticles facilitate the cellular uptake and promote cell death in the spheroid in vitro.

**Figure 6:**
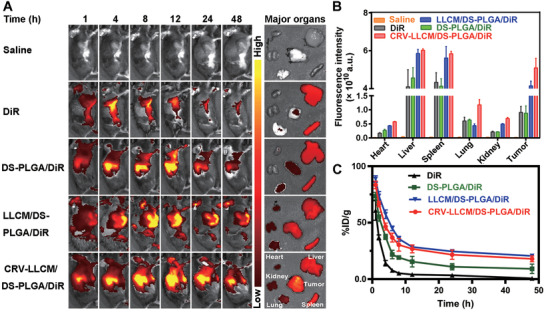
Biodistribution and circulation lifetime of different formulations in vivo. A) Fluorescence imaging of the biodistribution of different formulations (Saline, DiR, DS‐PLGA/DiR, LLCM/DS‐PLGA/DiR, and CRV‐LLCM/DS‐PLGA/DiR, respectively) in LLC‐allograft mice at a series of time points. B) Circulation lifetimes of different formulations (Saline, DiR, DS‐PLGA/DiR, LLCM/DS‐PLGA/DiR, and CRV‐LLCM/DS‐PLGA/DiR, respectively) at the indicated times. C) Quantitative biodistribution analysis of DiR‐labeled nanoparticles in major organs and tumors.

To assess the functional efficacy of CREATE in LLC‐allograft tumors, we analyzed the retention profiles and biodistribution using 1,1‐dioctadecyl‐3,3,3,3‐tetramethylindotricarbocyanine iodide (DiR) as an indicator in vivo. DiR itself diminished within 48 h, yet the membrane‐coated nanoparticles (LLCM/DS‐PLGA/DiR or CRV‐LLCM/DS‐PLGA/DiR) largely increased the retention time within the tumors as revealed by the fluorescence remaining at the time of 48 h (**Figure** **6**A). In terms of biodistribution, the bare DS‐PLGA/DiR was found to mainly localize in the liver and spleen, while the membrane‐coated nanoparticles were significantly distributed in the tumors (Figure 6A,B). The membrane‐camouflaged conferred the accumulation and retention of nanoparticles in the tumors. Further results found that the membrane‐coated nanoparticles exhibited a significantly prolonged circulation lifetime compared with the bare DS‐PLGA/DiR or DiR (Figure 6C). Of note, CRV‐LLCM/DS‐PLGA/DiR showed a comparable circulation performance with the LLCM/DS‐PLGA/DiR (Figure 6C). These results together suggest that the membrane camouflage greatly improves the specificity and circulation lifetime in vivo.

### 2.5 Suppressive Effect of dBET6‐Containing Formulations on Tumors

**Figure 7:**
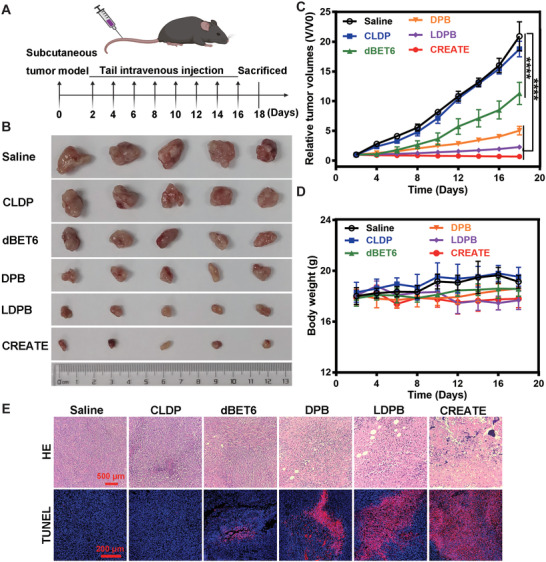
Inhibitory effects of different formulations in vivo. A) Schematic illustrating the in vivo therapeutic approach in the LLC tumor‐bearing mice. B) Gross morphology of tumors after intravenously inoculated with Saline, CLDP, dBET6, DPB, LDPB, and CREATE at the time of sacrifice (*n* = 5). C) Changes of relative tumor volumes detected at the indicated times. D) Changes in relative body weight detected at the indicated times. E) Representative images of HE and TUNEL staining of tumor sections treated with Saline, CLDP, dBET6, DPB, LDPB, and CREATE, respectively. Data are presented as mean ± SD. **** *p* < 0.0001.

To investigate the functional role of different formulations in suppressing tumor growth, we established the LLC cell derived tumor bearing mouse model (**Figure** **7**A). The administration with CREATE achieved the maximal tumor‐suppressive effects, which was 1/30 of the saline‐treated group (Figure 7B,C). In contrast, the treatment with CLDP, free dBET6, DPB, or LDPB showed much less therapeutic effect, corresponding to 1/1, 1/2, 1/4, and 1/9 of the saline‐treated group, respectively (Figure 7B,C). Meanwhile, no apparent changes were observed in the mice's body weight for all treatments (Figure 7D). In accordance with this, the HE staining of the liver, spleen, heart, kidney, and lung tissues showed no obvious inflammatory infiltrates or damage which indicated good biocompatibility of these formulations (Figure S18, Supporting Information). HE staining also revealed that CREATE induced more efficient tumor inhibition than treatment with LDPB (Figure 7E). In line with this, the TdT‐mediated dUTP Nick‐End Labeling (TUNEL) assay showed that LDPB and CREATE induced a significantly higher degree of apoptosis (Figure 7E and Figure S19, Supporting Information) than the control groups, such as saline‐, CLDP‐, dBET6‐, and DPB‐treated mice.

Inspired by the effective tumor inhibition by CREATE in the subcutaneous LLC tumor model, we further evaluated its effect on the orthotopic LLC tumor model. The orthotopic LLC tumor model was constructed by the in situ injection of the LLC cells into the left lung of C57BL/6 mice (Figure S20A, Supporting Information). We found that CREATE administration showed remarkable tumor inhibition compared with the control groups, such as saline and CLDP (Figure S20B, Supporting Information). About 90% mice survived over 18 d initially from the treatment with CREATE, when all of the mice were nearly dead in the control groups (Figure S20C, Supporting Information). Moreover, the total weight of lung tissue containing tumors was statistically one second‐fold lower in the CREATE group as compared with the control groups (Figure S20D, Supporting Information). The administration of CREATE showed minor changes in the body weight, implying its safety for in vivo use (Figure S20E, Supporting Information). Accordingly, HE staining revealed that CREATE induced more efficient tumor inhibition than treatment with saline and LDPB (Figure S21, Supporting Information). Collectively, the tumor‐suppressive experiments demonstrate that the dual‐targeting nano‐PROTAC enables effective tumor growth inhibition. In support of this, TAMs and LLC cells were eliminated significantly (Figure S22, Supporting Information). The infiltration of immune cells, including the CD4^+^ and CD8^+^ T cells, was significantly increased as evidenced by the immunofluorescence staining of the tumor sections (Figure S23, Supporting Information). The surrounding vessels were remarkably abolished, as a decrease of CD206, CD31, and *α*‐SMA was found (Figure S24, Supporting Information). These observations elucidate the underlying tumor‐suppressive mechanisms of CREATE, which is, by the restoration of local immune surveillance in the TME. Collectively, the tumor suppressive experiments demonstrate that the dual‐targeting nano‐PROTAC enables effective tumor growth inhibition with no systemic toxicity.

### 2.6 Remodeling of the TME by dBET6‐Containing Formulations

**Figure 8:**
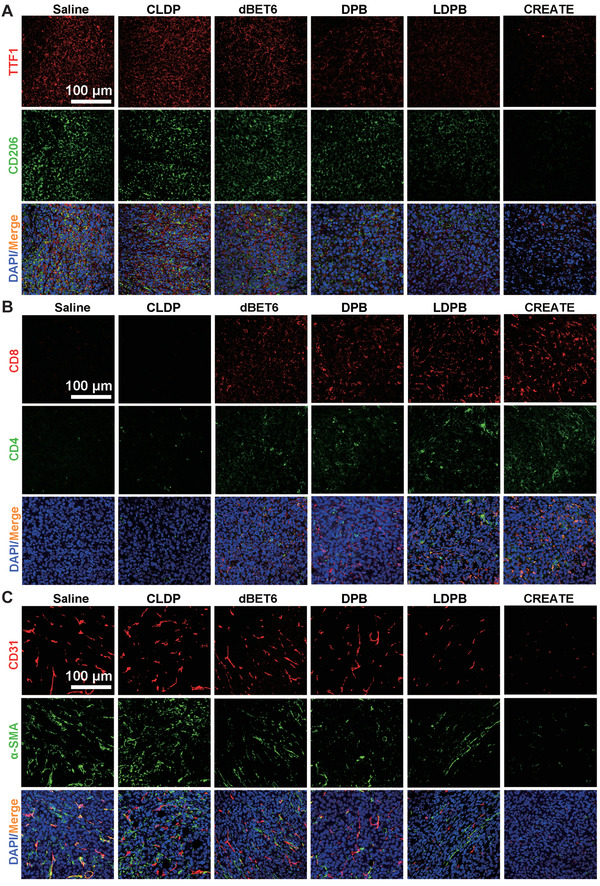
Effects of different formulations on the tumor microenvironment. A) Representative immunofluorescence staining of CD206^+^ and TTF1^+^ cells in the tumor tissues treated with PBS, CLDP, dBET6, DPB, LDPB, and CREATE. B) Representative immunofluorescence images of CD4^+^ and CD8^+^ T cell infiltration in the tumor tissues treated with Saline, CLDP, dBET6, DPB, LDPB, and CREATE. C) Representative immunofluorescence images of *α*‐SMA^+^ and CD31^+^ tumor blood vessels in the tumor tissues treated as above.

The TME has been widely implicated in tumorigenesis because it harbors tumor cells that interact with surrounding cells through the circulatory and lymphatic systems to influence the development and progression of cancer. Given the above role of CREATE in suppressing cancer cells and TAMs, we then investigated the remodeling of TME by CREATE. In agreement with cell‐based experiments, the immunofluorescence staining of the LLC‐allograft tumor section revealed a substantial decrease in TTF1 and CD206 expression levels, implying the effective elimination of LLC cells and TAMs (**Figure** **8**A). Depletion of M2‐like TAMs has been demonstrated to remodel the TME by activating the immune response.^[^ ^7^ ^]^ Accordingly, infiltration of immune cells, such as effector T cells (CD4^+^ and CD8^+^), was significantly increased as evaluated by the immunofluorescence staining of the tumor sections (Figure 8B). In addition, TAMs have been demonstrated to facilitate cancer progression by promoting angiogenesis, and that depletion of TAMs disrupt the secretion of angiogenic factors and blood vessel growth in tumors. We thus assessed the surrounding vessels by observing the colocalization of CD31 and *α*‐SMA, which are hallmarks of de novo vessels. Consistently, the administration of CREATE remarkably abolished the angiogenesis, as evidenced by the decrease of CD31^+^‐ and *α*‐SMA^+^‐stained cells (Figure 8C). Herein, our results propose a novel targeting strategy that effectively inhibits tumor growth, while facilitates the restoration of local immune surveillance in the TME, which in turn, regresses lung cancers.

## 3 Discussion

BRD4, as a transcriptional and epigenetic regulator, has recently emerged as a promising therapeutic target. The BRD4 inhibitor, JQ1, which blocks the ability of bromodomains to bind histones by competing for interaction with acetyl‐lysine motifs on histones, has been demonstrated to suppress lung cancer cell growth.^[^ ^8^ ^]^ In addition to this, JQ1 has also been reported to restrain the proliferation of TAMs through CSF1/CSF1R signaling. More recently, a series of BRD4 inhibitors have been developed and some of them (e.g., GSK525762/molibresib, NCT01587703; BMS‐986158, NCT03936465; AZD5153, NCT03205176) are entering early clinical trials for some tumors. However, their applications in the clinic are limited, likely due to relatively low potency, various toxicities, and low bioavailability. For instance, chronic in vivo use of BRD4 inhibitors has been associated with side effects, including reactivation of latent human immunodeficiency virus infection, immunosuppression, and some effects on the central nervous system.^[^ ^24^ ^]^ Accordingly, several approaches have been utilized to improve their potency, including the development of novel BET degrader based on heterobifunctional Proteolysis targeting chimera (PROTAC) technology.^[^ ^12a^ ^]^ Basically, a BET inhibitor was linked to another small‐molecule ligand that binds the cereblon (CRBN) E3 ubiquitin ligase complex, thereby causing significant degradation of BRD4 by the proteosome system. In 2015, BRD4 was reported as the first epigenetic protein applied to chemically induced protein degradation. Later, a series of dBET1, ARV‐825, and MZ1 were identified by different groups, and all relied on the structure of JQ1 as a BRD4 binding ligand.^[^ ^25^ ^]^ dBET6 as a new generation of BRD4 PROTAC, shows better performance in BRD4 degradation. The development of dBET6‐based formulations may contribute to efficient cancer therapy by BRD4 degradation, which exhibits a more profound effect independent of its catalytic activity on gene transcription and cell growth regulation than BRD4 inhibition while enabling lower drug exposure and less frequent dosing.^[^ ^13^, ^26^ ^]^

Cell membranes derived from red blood cells, platelets, leukocytes, macrophages, and cancer cells have been utilized to ameliorate the material interface and mimic cellular functions by fusing natural cell membranes onto synthetic cores.^[^ ^27^ ^]^ For instance, we have recently demonstrated that membranes derived from lung cancer cells significantly enhanced the therapeutic efficiency and delivered specificity of siRNAs or epigenetic inhibitors to cancer cells via homotypic targeting.^[^ ^28^ ^]^ Herein, an optimized BET degrader, dBET6, was incorporated into CRV‐LLCM to improve their low solubility, poor permeability, and limited pharmacokinetics for dual‐targeting of cancer cells and TAMs. In agreement with our previous findings,^[^ ^28^, ^29^ ^]^ the present study revealed that the CRV‐engineered lung cancer cell membranes possess homotypic targeting ability, for instance, simultaneous targeting lung cancer cells and TAMs for apoptosis both in vitro and in vivo; this findings provide a novel strategy for regressing lung cancer.

In addition, stimuli‐responsive nanoparticles are gaining significant insight for treating various cancers that dispense different endogenous stimuli, including light, magnetic field, ultrasound, pH, temperature, enzyme, redox potential, etc., to trigger the drug release from these nanoparticles.^[^ ^30^ ^]^ Among these reported stimuli, redox potential has attracted considerable attention due to the uniquely heterogeneous redox potential gradient in the tumor site.^[^ ^31^ ^]^ The extracellular matrix is oxidative as a result of the overproduction of ROS, while the intracellular cytoplasm is reductive because of a relatively high level of glutathione (GSH) in most tumor sites. The GSH levels in the intracellular environment of cancer cells or M2‐like macrophages are over 100–1000 times (around 2–10 × 10^−3^ m) higher than that in the environment surrounding these cells.^[^ ^32^ ^]^ Disulfide bond is a kind of dynamic covalent bond widely existing in organisms. In normal body fluid circulation, disulfide bonds can exist stably due to their high bond energy (240 kJ mol^−1^). However, the disulfide bond will be reduced to the sulfhydryl group due to the high level of GSH within tumor cells, and then the molecular chain containing the disulfide bond will be cleaved, thus can be utilized for the construction of GSH‐responsive drug delivery systems to achieve the enhanced therapeutic efficacy against tumors by releasing the payloads.^[^ ^33^, ^34^ ^]^ In this study, DS‐PLGA is linked by disulfide bond between PLGA, and GSH acts as a reducing agent to break disulfide bonds, forming oxidized glutathione and the disulfide bonds are reduced to sulfhydryl groups (—SH), so as to achieve a stimulated release of payloads. The in vitro drug release analysis demonstrated that the GSH‐sensitive property enabled the nanostructure to release the drugs about 60% until 72 h under a GSH environment. Regarding the pH sensitivity of DS‐PLGA, PLGA was first polymerized by polylactic acid and polyglycolic acid and then was linked with an ester bond that is sensitive to the acidic environment. It is well‐known that there is a lower pH in the organelles, such as endosomes and lysosomes (pH 5.0–6.5), inside the cells.^[^ ^35^ ^]^ The pH‐responsive DS‐PLGA was also stimulated to release drugs in the acidic tumor or endosome/lysosome microenvironments. As expected, the in vitro drug release demonstrated that the pH‐sensitive property improved the release of dBET6 significantly. Thus, we have designated pH/GSH‐responsive nanoparticles, which greatly improve the stimulated release of PROTAC in the target cells.

TAMs are a major type of infiltrating immune cells in lung cancer that acts as a source of local and systemic cues to support the proliferation, survival, and migration of tumor cells. Our strategy not only inhibits tumor “seeds,” but also renovates the tumor “soil” to construct a tumor‐suppressive microenvironment, e.g., activating T cells and suppressing angiogenesis. Our results have found that CREATE simultaneously targeted cancer cells and their surrounding TAMs to apoptosis, at least in part via a caspase‐3‐dependent manner. This is supported by a recent study in which a new BRD4‐targeting degrader was identified to selectively increase the expression of cleaved Poly (ADP‐ribose) polymerase‐1 (PARP) and apoptosis in treatment‐refractory solid lung cancer.^[^ ^36^ ^]^ It was also demonstrated that BET degraders such as ZBC260 and dBET can effectively induce Bim‐dependent apoptosisof human lung cancer cells via the suppression of Mcl‐1 and c‐FLIP.^[^ ^18^ ^]^ Thus, it will be interesting to further explore the underlying mechanism of CREATE in controlling cancer cells and TAMs apoptosis. On the other hand, we are the first to use BRD4 PROTAC‐based nanoparticles to remodel TME and activate the immune response, which facilitated the infiltration of effector CD4^+^ and CD8^+^ T cells. The above evidence demonstrates that this style of versatile nano‐PROTAC holds great potential for efficient lung cancer therapy.

## 4 Conclusion

In summary, we have developed a versatile nano‐PROTAC that exhibits dual‐targeting ability of lung cancer cells and TAMs by the camouflage with CRV‐overexpressing LLCM. The tumor‐suppressive effects are attributed by the induction of LLC cells via a caspase‐3‐dependent apoptosis and directly killing of TAMs, which in turn, remodels the TME. Furthermore, simultaneous targeting cancer cells and the tumor growth‐supporting TAMs is a promising strategy to remodel the TME. Our findings suggest a novel strategy for lung cancer therapy involving engineered cell membranes to efficiently deliver the BRD4 PROTAC for the dual‐targeting of cancer cells and TAMs.

## 5 Experimental Section

### Cell Culture and M2 Polarization

LLC and HEK 293T cells were purchased from the Cell Resources Center of Shanghai Institutes for Biological Sciences, Chinese Academy of Sciences (SIBS, CAS, China). LLC cells were cultured in Dulbecco's modified Eagle's medium (DMEM) supplemented with 10% fetal bovine serum (FBS, Gibco, USA), 100 U mL^−1^ penicillin, and 100 U mL^−1^ streptomycin. The murine macrophage cell line Raw 264.7 was maintained in DMEM containing 10% FBS plus 100 U mL^−1^ penicillin‐streptomycin mixed solutions. For M2 polarization, Raw 264.7 cells at a density of 5 × 10^4^ cells/well were seeded into six‐well plates, followed by the treatment with IL‐4 (50 ng mL^−1^, MedChemExpress) for 2 d. M2 polarization upon induction was detected by flow cytometry with anti‐F4/80 and anti‐CD206 antibodies (BD Bioscience, Cat. No. 565410 for PE Rat Anti‐Mouse F4/80 and Cat. No. 565250 for Alexa Fluor 647 Rat Anti‐Mouse CD206). All cell lines were incubated at 37 °C in a 5% CO_2_ atmosphere.

### Biochemicals and Antibodies

PLGA‐S‐S‐PLGA (DS‐PLGA) polymer materials (purity, 90%) were purchased from Xi'an Ruixi Biological Technology Co., Ltd. (Xi'an, China). The primary antibodies used in the present study included anti‐BRD4 (1:2000, BETHYL, USA), anti‐c‐Myc (1:1000, CST, USA), anti‐caspase 3 (1:1000, CST, USA), anti‐Cleaved‐caspase 3 (1:1000, CST, USA), anti‐Lamp2 (1:1000, Abcam, USA), and anti‐TTF1 (1:1000, Abcam, USA). DiR (purity > 98%), DiD (purity > 98%), fluorescein isothiocyanate (FITC, purity > 95%), and 2‐(4‐amidinophenyl)‐6‐indolecarbamidine dihydrochloride (DAPI, purity > 98%) were obtained from Thermo Fisher Scientific Inc. (Waltham, USA). The GSH assay kit was provided by Beyotime (Shanghai, China).

### Preparation of dBET6‐Loaded Nanoparticles (DPB)

PLGA‐S‐S‐PLGA (DS‐PLGA) was dissolved into a solution comprised of ethyl acetate and dichloromethane (v/v = 3/7), and dBET6 (dissolved in dichloromethane) was thoroughly mixed with the solution of DS‐PLGA at different weight ratios (DS‐PLGA/dBET6 = 5/1, 10/1, 15/1, 20/1, 30/1, 40/1, and 50/1). Ultrasonic treatment was performed in an ice water bath (Power 180 W, 3 s on and 1 s off for 5 min). Next, the obtained emulsion was dropped into a 3% polyvinyl alcohol (PVA) solution under ultrasonic treatment. Finally, the organic solvent was volatilized dried by a rotary evaporator, and the precipitate was collected by centrifugation at 12 000 *g* at 4 °C for 40 min. The residual surfactant was washed twice with PBS, and the precipitate was collected by centrifugation and resuspended in PBS for later use.

### Construction of CRV‐Overexpressing LLC Cell Lines

A peptide (amino acid sequence, CRVLRSGSC, abbreviated as CRV) that selectively bound to TAMs was previously described.^[^ ^16b^ ^]^ A total of 8 × 10^6^ HEK 293T cells were plated in 15‐cm plates 24 h before transfection so that the cells were 30%–50% confluent when transfected. For one 15‐cm plate transfection, the following four DNAs, including 10 µg CRV‐Lamp2 fusion plasmid, 5 µg pVSV‐G, 5 µg pRSV‐REV, and 5 µg pMDL g/p RRE, were mixed into HEK 293T cells following the standard Lipofectamine transfection protocol. The medium (which now contains viral particles) was collected 48 h after transfection, and another 24 h (72 h after transfection) was waited to conduct the second harvest. The collected medium was centrifuged at 3000 rpm for 15 min at room temperature to pellet the cell debris. The virus‐containing supernatants were transferred into a new tube, and supernatants were filtered through a 0.45 µm filter with a syringe. The supernatants were next centrifuged at 19 400 rpm, 4 °C for 2 h to obtain the concentrated virus, which was diluted with PBS. The purified virus suspension was added to the culture medium of LLC cells for 72 h, and immunoblotting assays were conducted to examine the CRV‐Lamp2 expression using antibody against Lamp2.

### Construction of CREATE

A cell membrane isolation kit (Beyotime, China) was used to separate CRV‐LLCM according to the manufacturer's instructions. The membranes were mixed with DPB with different weight ratios (CRV‐LLCM/DPB = 1/1, 2/1, 3/1, 4/1, and 5/1), and the mixture was continuously vortexed for 5 min and ultrasonicated for 3 min. Next, a Miniextruder (Avanti Polar Lipids, Inc., Alabaster, USA) was used to successively coextrude the mixture with 400, 200, and 100 nm polycarbonate films 15 times back and forth to obtain CRV‐LLCM‐coated DPB. As a control, LDPB was prepared using LLCM to substitute the CRV‐LLCM with the same procedure.

### Characterization of Prepared Nanoparticles

Different proportions of materials were measured at room temperature using Zeta Sizer (Malvern, Worcestershire, UK), including the particle size, zeta potential, and PDI. Another batch of prepared DPB and CREATE were exposed to PBS solution at various time courses to evaluate their stability. 10 µL of diluted DPB and CREATE solutions were placed onto a copper grid for 3–5 min, followed by the absorption of filter paper, which was dried overnight and observed by TEM (JEOL, Tokyo, Japan). Referring to the previous work,^[^ ^37^, ^38^, ^39^ ^]^ the changes of nanoparticles in the presence of acidic and/or GSH environments were also analyzed. DPB was incubated in the conditions, such as PBS (pH 7.4), pH 6.5, pH 5.0, GSH 5 × 10^−3^ m, GSH 10 × 10^−3^ m, pH 6.5/GSH 5 × 10^−3^ m, pH 6.5/GSH 10 × 10^−3^ m, pH 5/GSH 5 × 10^−3^ m, pH 5/GSH 10 × 10^−3^ m for 6 h. The suspension was dropped onto the copper grid and air‐dried overnight prior to the TEM analysis.

To determine the drug loading capacity and encapsulation efficiency, the standard curve was first established by the ultraviolet (UV) spectrophotometer, followed by calculation with the following formulas

(1) $$\mathrm{Drug}\;\mathrm{loading}\;\mathrm{capacity}\;\left(\%\right)=\left(W_{i}-W_{s}\right)/W_{t}\times 100\%$$

(2) $$\mathrm{Encapsulation}\;\mathrm{efficiency}\;\left(\%\right)=\left(W_{i}-W_{s}\right)/W_{i}\times 100\%$$

where *W*_s_: drug content of supernatant; *W*_i_: initial input of drugs; and *W*_t_: total weight of nanoparticles.

### In Vitro Drug Release Test

The prepared nanoparticles were dissolved in 1 mL double‐distilled water (ddH_2_O) and then placed into a treated dialysis bag. The dialysis bag was placed in a centrifuge tube containing 15 mL PBS at a speed of 120 r min^−1^ in a constant temperature shaker at 37 °C. 3 mL of liquid were taken at a predetermined time point to measure UV absorption to further calculate the drug content of the removed liquid. The cumulative drug release was calculated, and the in vitro drug release curve was plotted.

### Cellular Uptake Assay

CLSM and FACS analyses were used to determine the cellular uptake efficiency and the localization of nanoparticles in LLC cells and M2‐like macrophages. Due to the lack of fluorescence, dBET6 was replaced by 1,1′‐dioctadecyl‐3,3,3′,3′‐tetramethylindodicarbocyanine, 4‐chlorobenzenesulfonate salt (DiD), which was loaded into nanoparticles. The cells were first inoculated in confocal dishes and six‐well plates with different nanoparticle volumes or predetermined times (3, 6, 9, and 12 h). After the specified culture time, the medium was removed, and the cells were washed with PBS thrice and fixed with 4% paraformaldehyde at 37 °C for 10 min, followed by culturing with 0.1% Triton for 10 min. Then, phalloidin‐Alexa Fluor 555 was used to stain the cytoskeleton for 50 min, and DAPI was used to stain the cell nucleus for 20 min. The cells were observed by CLSM (Zeiss 880, Germany). Flow cytometry was used for fluorescence quantification assessment. Cells were collected after digestion with trypsin. After two rounds of centrifugation with PBS, the cells were resuspended in 30 µL PBS, and fluorescence was quantified by flow cytometry (BD, San Jose, CA).

To investigate the internalization mechanism, the LLC cells or M2‐like macrophages were pretreated with the medium containing CPZ (Cayman Chemical, USA), GEN (Sigma‐Aldrich, USA), or AMI (Selleck, USA) for 2 h before the administration with CREATE. Alternatively, the cells were also placed at 4 °C to study the effect of temperature on the internalization of CREATE individually. The medium was replaced by freshly prepared medium containing CRV‐LLCM/DS‐PLGA/DiD and then incubated for another 4 h at 37 °C with 5% CO_2_ atmosphere, followed by the analysis with FACS and CLSM.

### Apoptosis Assay

A total of 5 × 10^4^ cells were cultured in six‐well plates for 24 h, then the cells were treated with different formulations (PBS, CLDP, dBET6, DPB, LDPB, and CREATE, respectively) for 9 h, and incubated with the freshly prepared medium for another 15 h. The apoptotic rate was measured with Annexin V‐FITC/PI Apoptosis Detection Kit (Thermo Fisher Scientific Inc., USA), then quantified by flow cytometry (BD, San Jose, CA).

### Live and Death Staining Assay

A total of 5 × 10^4^ cells were inoculated in confocal dishes for 24 h. Each well was treated with different groups as mentioned above for 9 h, and incubated with the freshly prepared medium for another 15 h, and the culture medium was removed and replaced with the newly prepared medium. The cells were cultured with the Live/Dead staining kit at 4 °C for 30 min under dark conditions. After washing with PBS, the cells were observed under CLSM.

### Colony Formation Assays

A total of 500 cells LLC cells were inoculated in six‐well plate for 24 h. The cells were treated with different formulations (PBS, CLDP, dBET6, DPB, LDPB, or CREATE, respectively) for 9 h, and incubated with the freshly prepared medium for another 7 d. The cells were fixed with 4% paraformaldehyde at 37 °C for 15 min. Then, 0.1% crystal violet solution was used to stain the cells for 10 min. The cells were washed and imaged with a camera.

### Spheroid Preparation and Handling

To obtain the M2/LLC spheroids, a density of 8 × 10^3^ LLC cells and M2‐like macrophages (4 × 10^3^ LLC cells and 4 × 10^3^ M2‐like macrophages, respectively) were jointly seeded into 96‐well culture plates, which were previously covered with 2% agarose. After the cells adhered to the plate, the 96‐well culture plate was moderately shaken in an orbital direction and then incubated for 5 d until spheroids were formed. For cellular uptake detection, the medium was replaced by a fresh medium supplemented with DS‐PLGA/DiD, LLCM/DS‐PLGA/DiD, and CRV‐LLCM/DS‐PLGA/DiD. Spheroids were washed thrice with PBS and fixed with 4% paraformaldehyde at 37 °C for 15 min. Then, DAPI was used to stain the cell nuclei of spheroids for 0.5 h. For the Live/Dead staining assay, the medium was replaced by the fresh medium supplemented with different groups (PBS, CLDP, dBET6, DPB, LDPB, and CREATE) for 9 h, and incubated with the freshly prepared medium for another 15 h. The cells were cultured with the Live/Dead staining kit at 4 °C for 30 min under dark conditions. After washing with PBS, the spheroids were observed by confocal laser microscopy (Zeiss 880, Germany). About the LLC spheroids, they were prepared according to the procedure described above, except for the single use of LLC cells.

### Immunoblotting

LLC cells were treated with different drug formulations (PBS, CLDP, dBET6, DPB, LDPB, and CREATE, respectively) for 9 h, and incubated with the freshly prepared medium for another 15 h. The total cell lysate was prepared as described previously.^[^ ^14a^ ^]^ In brief, aliquots of each sample containing 20 µg of protein were separated by electrophoresis using SDS‐PAGE gels (Beyotime, Cat. No. P0012A, China), and transferred to polyvinylidene fluoride membranes (PVDF, PALL, Cat. No. BSP0161, MA, USA). After blocking with nonfat dry milk for 1 h at room temperature, the membranes were probed and imaged with primary and secondary antibodies as mentioned above. Tubulin was used as an internal control.

### Animal Experiments

The female C57BL/6 (weight 18–20 g) were purchased from Guangdong Medical Lab Animal Center and raised in the Specific pathogen‐free laboratory animal room. All of the protocols for the animal experiments were approved by the Institutional Animal Care and Use Committee of Guangzhou Medical University (Approval No. GY2019‐149).

### Circulation Lifetime

The C57BL/6 mice were randomly divided into four groups (DiR, DS‐PLGA/DiR, LLCM/DS‐PLGA/DiR, and CRV‐LLCM/DS‐PLGA/DiR). Then, the mice were intravenously injected with different formulations (DiR equivalent to 0.1% of DS‐PLGA). The whole‐body fluorescence distribution patterns at 1, 2, 4, 6, 8, 12, 24, and 48 h were measured using the collected blood samples by RF‐6000 fluorescence spectrophotometer (Shimadzu, Japan) described previously.^[^ ^15a^ ^]^ DiR blood solution was diluted to 20, 15, 10, 5, 2.5, 1, and 0.5 µg mL^−1^ to establish the DiR standard curve.

### Biodistribution of Nanoparticles by In Vivo Imaging System

The LLC cells were subcutaneously injected into the armpits of the flanks of C57BL/6 mice. After the tumors grew to ≈200 mm^3^, the LLC tumor‐bearing mice were randomly divided into five groups: saline, DiR, DS‐PLGA/DiR, LLCM/DS‐PLGA/DiR, and CRV‐LLCM/DS‐PLGA/DiR. The formulations were intravenously injected into mice (DiR equivalent to 0.1% of DS‐PLGA). The mice were anesthetized with 2% isoflurane, and their distribution was monitored at pointed times (1, 4, 8, 12, 24, and 48 h, respectively) by an imaging system (IVIS Lumina XRMS Series III, PerkinElmer, USA). After euthanasia of the mice, the dissected tumors and major organs (heart, liver, spleen, lung, and kidney) were collected for ex vivo imaging. The fluorescence intensity of the isolated tumors and organs was quantified using Living Image (64‐bit) software.

### In Vivo Tumor Inhibition Assay

The tumor inhibition induced by CREATE in the subcutaneous LLC tumor model was first evaluated. The LLC cells were subcutaneously injected into the armpits of the flanks of C57BL/6 mice. After the tumors grew to ≈50 mm^3^, the LLC tumor‐bearing mice were randomly divided into six groups: saline, CLDP, dBET6, DPB, LDPB, and CREATE. Different formulations were intravenously injected into mice every 2 d, and the mice were sacrificed on day 19. The dBET6‐containing formulations were administrated to the LLC cell tumor‐bearing C57BL/6 mice at a dosage of 5 mg kg^−1^ via intravenous injection referred to the previous work. During this period, tumor volumes were measured using caliper measurements and calculated as follows: tumor size (mm^3^) = (width^2^ × length)/2. At the end of the experiment, the mice were weighed and sacrificed, and the tumor tissues and main organs, including the heart, liver, spleen, lung, and kidney, were collected for further analyses.

The in vivo orthotopic LLC model was utilized to investigate the therapeutic effect of CREATE on the treatment of lung cancer. Briefly, the female C57BL/6 (weight 18–20 g) mice were purchased from Guangdong Medical Lab Animal Center. To establish the orthotopic LLC model, mice were anesthetized with pentobarbital at a dosage of 50 mg kg^−1^ through intraperitoneal injection. A total of 50 µL mixture containing LLC cells (2 × 10^6^ mL^−1^) and Matrigel were injected into the left lung of mice. After 7 d, the orthotopic LLC tumors were confirmed after the dissection of mice from the same batch. The orthotopic LLC tumor‐bearing mice were randomly divided into three groups including saline, CLDP, and CREATE, respectively. Then, the mice were intravenously injected with different formulations every 3 d. After six injections, the mice were sacrificed and the lung tissues were extracted for further analysis.

### Immunofluorescence Analysis of Tumor Tissues

The tumor tissue sections were deparaffinized, rehydrated, and placed in a repair box filled with EDTA antigen repair buffer (pH 8.0) for antigen repair. Then, the objective tissues were added with 3% BSA to cover the marked tissue to block non‐specific binding for 30 min and covered with 10% donkey serum (for the case of primary antibodies originating from goats). The sealing solution was gently removed, and PBS was added to the sections by dropping the primary antibodies (anti‐CD206, anti‐TTF1, anti‐CD4, anti‐CD8, anti‐SMA, anti‐CD31) prepared in a certain proportion. The sections were placed horizontally in a wet box at 4 °C for incubation overnight and covered with secondary antibody, incubating at room temperature for 50 min in dark conditions, followed by incubating with DAPI solution at room temperature for 10 min. The slices were briefly shaken dry and sealed with anti‐fluorescence quenching sealing tablets to conduct microscopy detection (Zeiss 880, Germany).

### Statistical Analysis

Three independent experiments were carried out for each analysis unless stated otherwise. Continuous variables were expressed as the mean ± standard deviation (SD). The statistical significance between the two groups was measured using the unpaired Student's *t*‐test. Statistical differences are shown as *p < 0.05, **p < 0.01, and ***p < 0.001.

## Conflict of Interest

The authors declare no conflict of interest.

## Author Contributions

H.T.Z., R.P., and S.C. contributed equally to this work. L.M.Z.: conceptualization, supervision and H.T.Z.: writing‐original draft, writing‐review and editing.

## Supporting information

Supporting Information

## Data Availability

The data that support the findings of this study are available from the corresponding author upon reasonable request.
