# Supplementary material for: Versatile Nano‐PROTAC‐Induced Epigenetic Reader Degradation for Efficient Lung Cancer Therapy
Source: Adv Sci (Weinh). 2022 Aug 21;9(29):2202039. doi: 10.1002/advs.202202039 (PMC9561860; doi:10.1002/advs.202202039)
Supplement: Supplementary file 1 — Supporting Information [file ADVS-9-2202039-s001.pdf]

ADVANCED SCIENCE
Open Access

Supporting Information

for *Adv. Sci.*, DOI 10.1002/advs.202202039

Versatile Nano-PROTAC-Induced Epigenetic Reader Degradation for Efficient Lung Cancer Therapy

Huan-Tian Zhang, Rui Peng, Sheng Chen, Ao Shen, Lixin Zhao, Wang Tang, Xiao-He Wang, Zhen-Yan Li, Zhen-Gang Zha, Mengmeng Yi* and Lingmin Zhang**

Supporting information

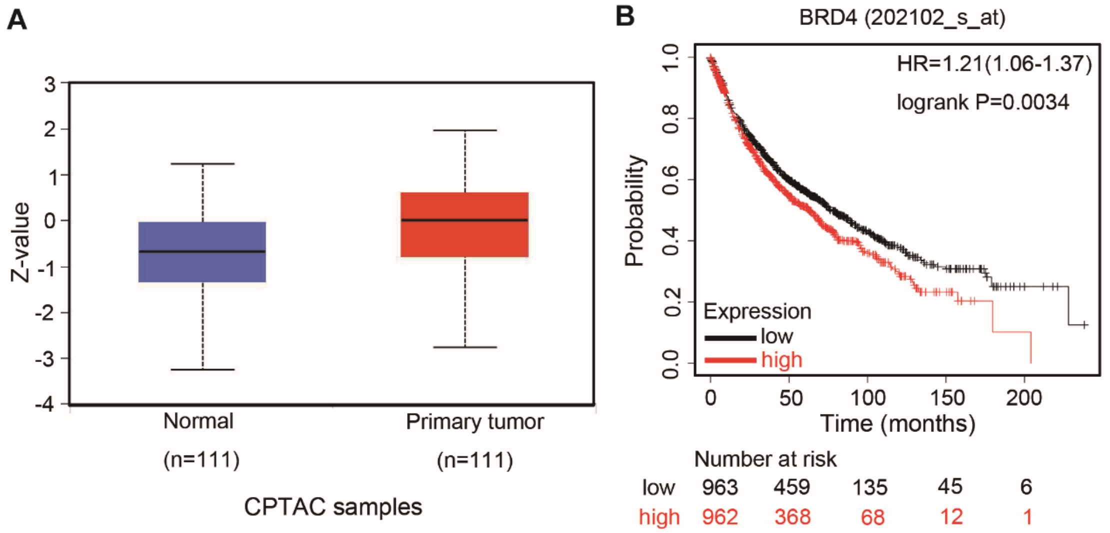

Figure S1. The bioinformatic analysis of BRD4 expression in lung cancer. (A) Protein expression level of BRD4 between normal lung tissues and lung adenocarcinoma was explored using the CPTAC (clinical proteomic tumor analysis consortium) database. (B) The correlation of probability with different levels of BRD4 was analyzed using Kaplan–Meier survival curve.

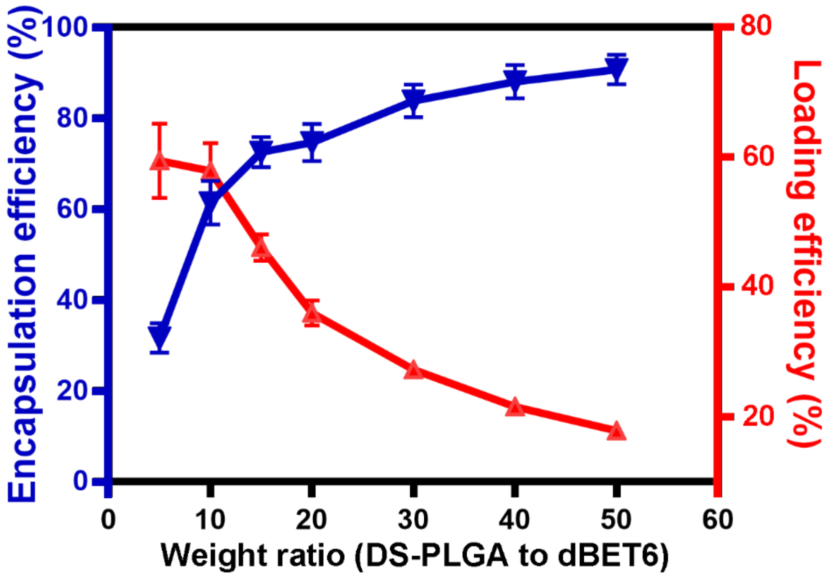

Figure S2. The encapsulation efficiency (%) and loading efficiency (%) with different weight ratios of DS-PLGA to dBET6 (5/1, 10/1, 15/1, 20/1, 30/1, 40/1, and 50/1, respectively) (n=3).

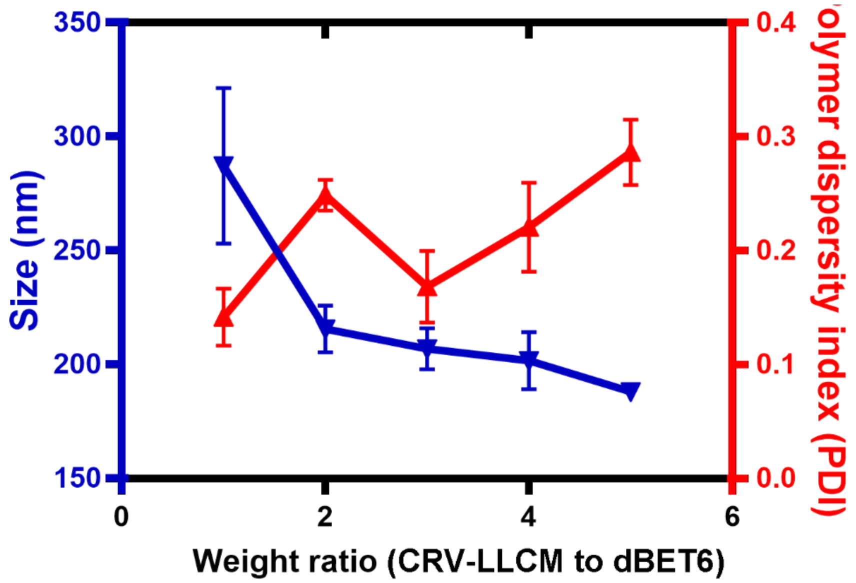

Figure S3. The size and polymer dispersity index (PDI) of CREATE with different weight ratios of CRV-LLCM to DPB (1/1, 2/1, 3/1, 4/1, and 5/1, respectively) (n=3).

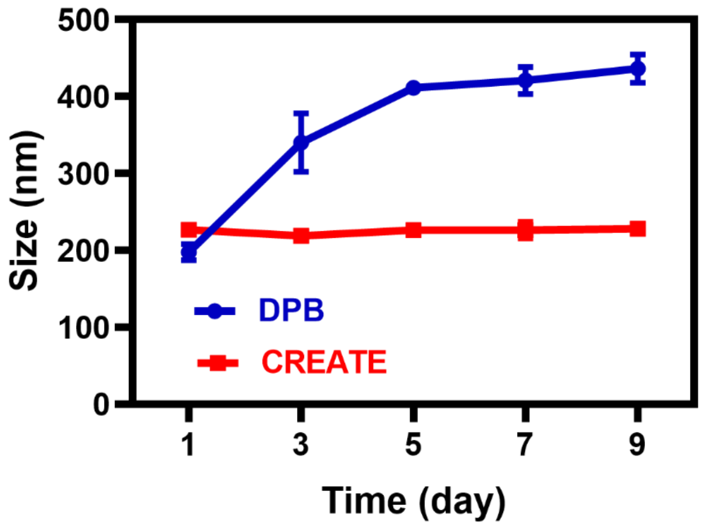

Figure S4. The stability of DPB and CREATE at different times in PBS (n=3).

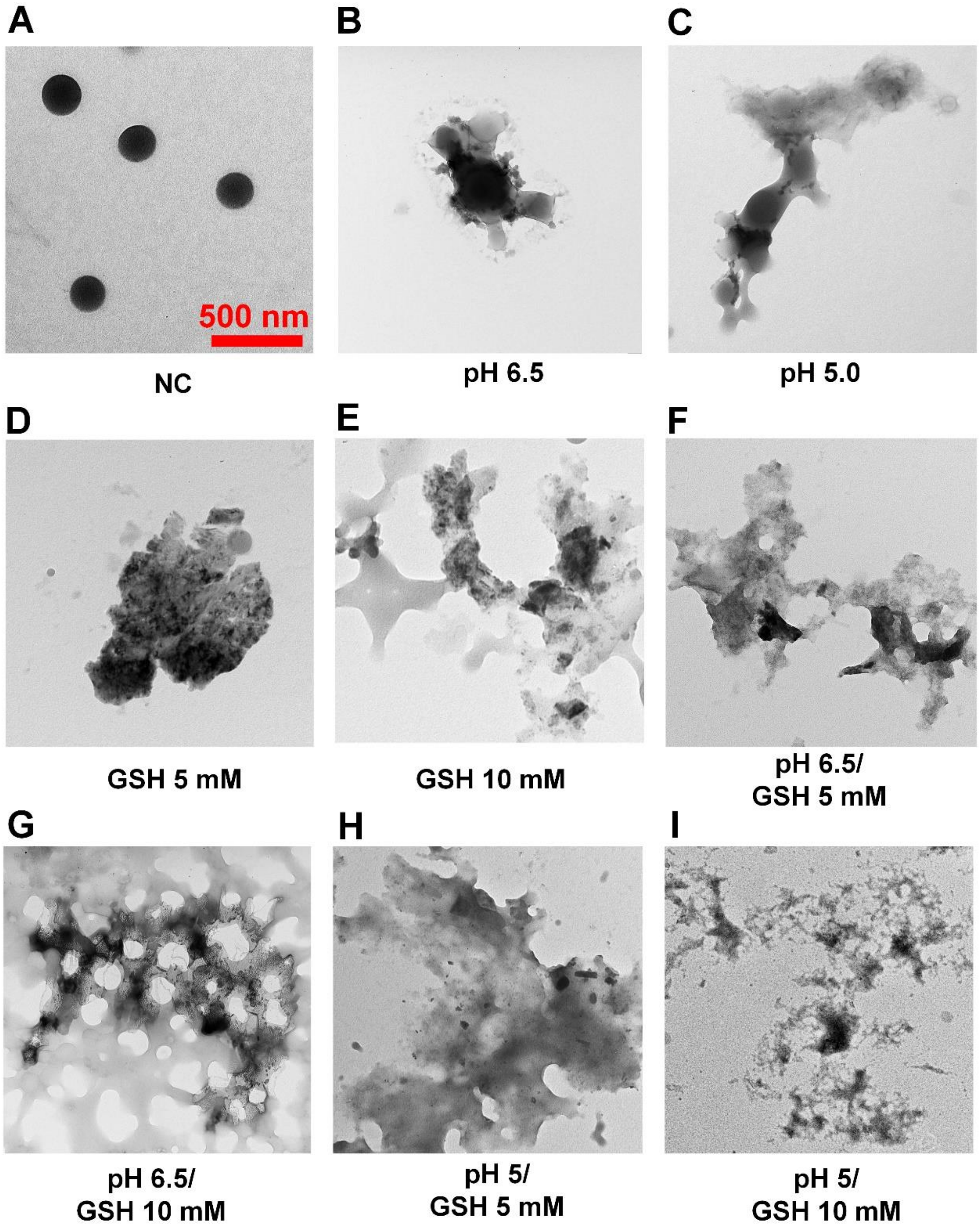

Figure S5. TEM analysis of the morphology of DPB. TEM was used to visualize the morphological change of DPB nanostructure cultured under different pH or GSH environments for 6 h. A, B, and C) Representative TEM images of DPB after 6 h of incubation at pH 7.4, 6.5, or 5.0, respectively; D, E) Representative TEM images of DPB after 6 h of incubation at GSH 5 mM or 10 mM, respectively; F, G, H, and I) Representative TEM images of DPB after 6 h of incubation at pH 6.5, or 5.0, combined with GSH 5 mM or 10 mM.

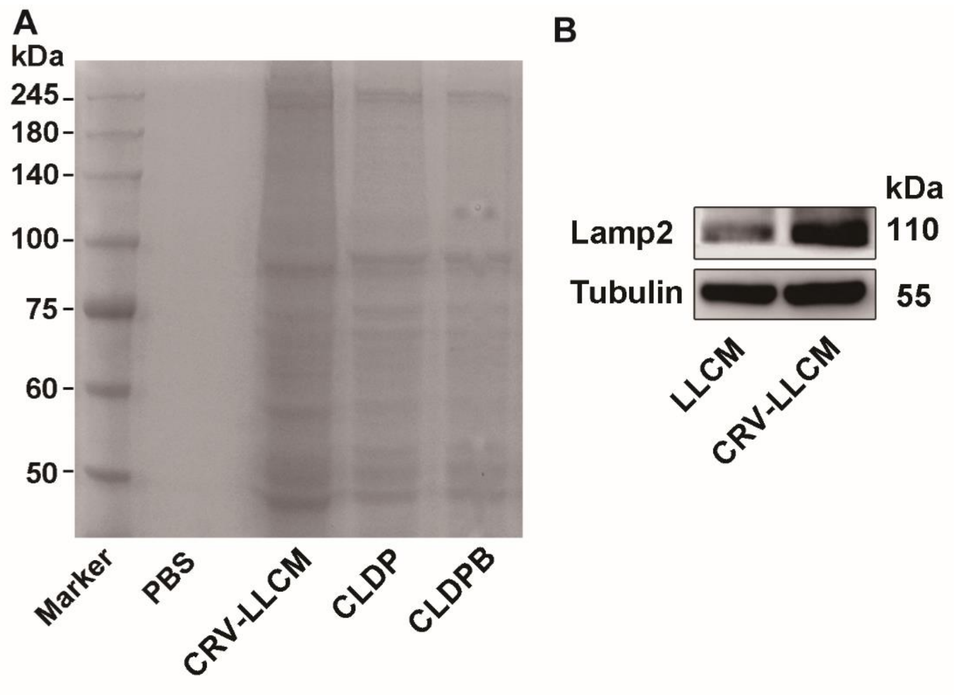

Figure S6. Characterization of CRV-LLCM-coated nanoparticles. (A) SDS-PAGE analysis of the protein composition of different formulations. (B) Detection of Lamp2-CRV fusion protein using the antibody against Lamp2 by immunoblotting.

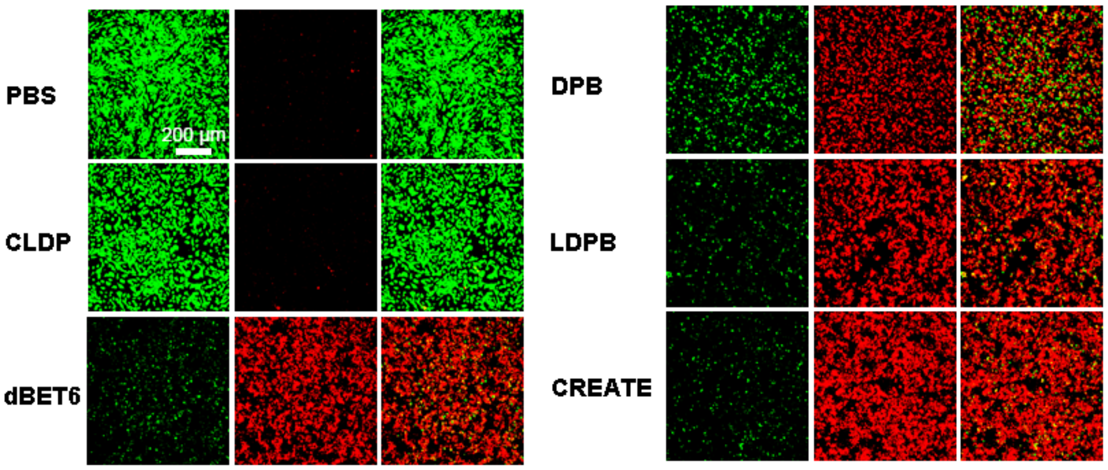

Figure S7. Live/Dead assay analysis of LLC cells treated with different formulations (PBS, CLDP, dBET6, DPB, LDPB, and CREATE, respectively) for 9 h, and incubated with the freshly prepared medium for another 15 h.

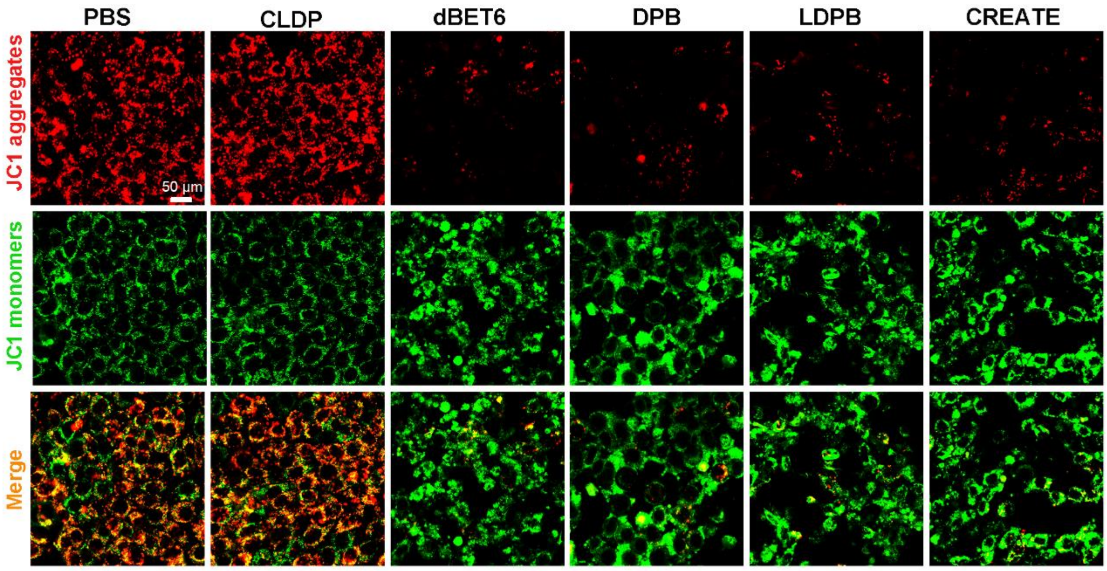

Figure S8. Mitochondrial membrane potential detection of LLC cells treated with different formulations (PBS, CLDP, dBET6, DPB, LDPB, and CREATE, respectively) for 9 h, and incubated with the freshly prepared medium for another 15 h, followed by the staining with JC1 kit.

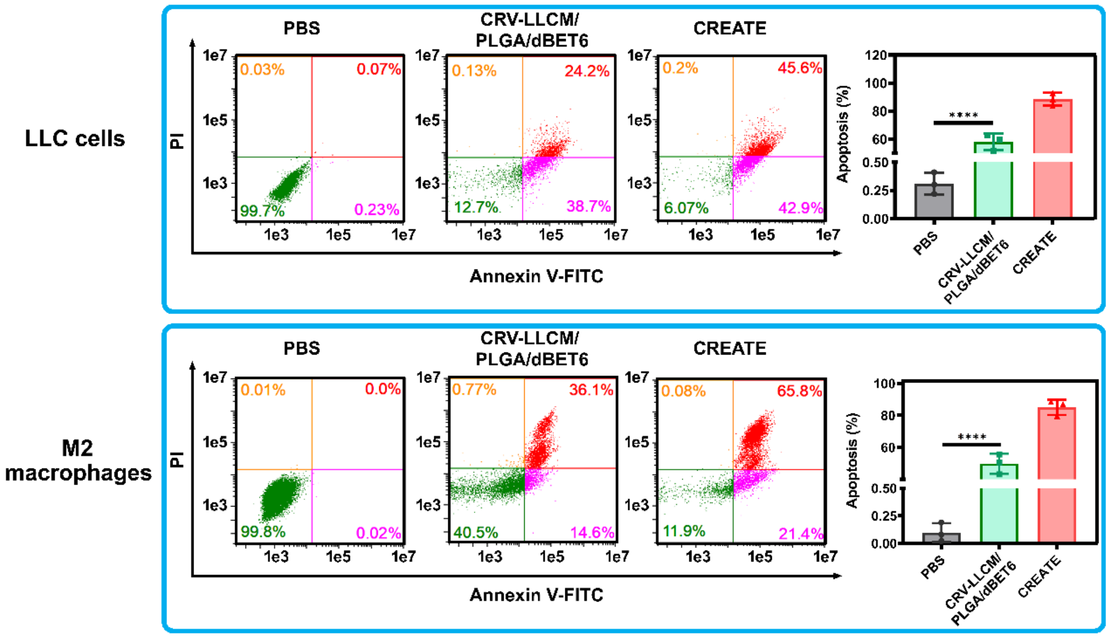

Figure S9. Quantitative analysis of cell apoptosis induced by different formulations. Data are presented as mean ± SD. Statistically significant differences were demonstrated between different samples and CREATE. **** $p < 0.0001$. CRV-LLCM/PLGA/dBET6 was defined as pH-responsive but GSH-nonresponsive materials (PLGA) control, while CREATE was constructed with pH/GSH-responsive materials (DS-PLGA) (n=3).

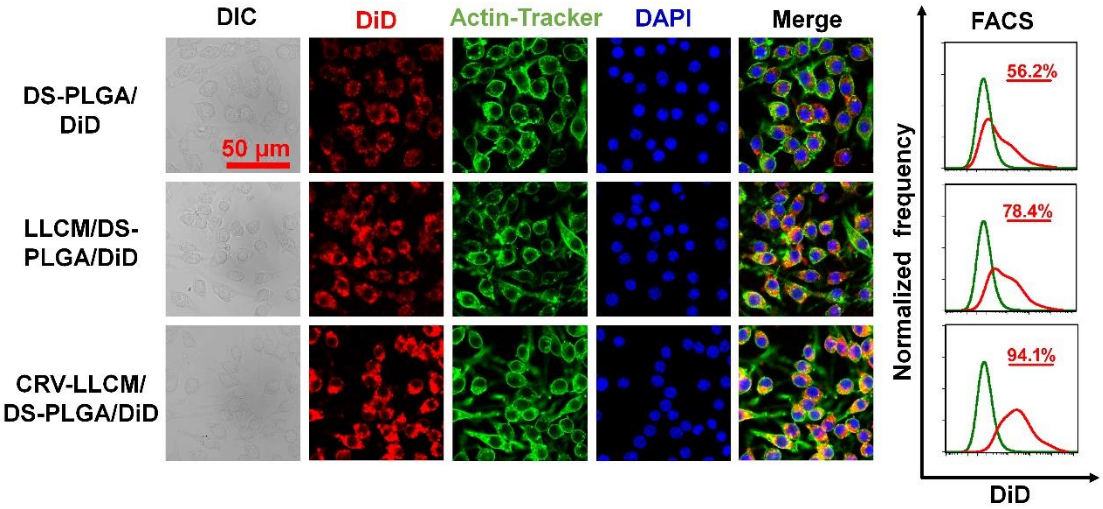

Figure S10. Cellular uptake of DS-PLGA/DiD, LLCM/DS-PLGA/DiD, and CRV-LLCM/DS-PLGA/DiD. M2-like macrophages were treated with the indicated nanoparticles (DS-PLGA/DiD equivalent to 60 μg/mL; LLCM/DS-PLGA/DiD and CRV-LLCM/DS-PLGA/DiD equivalent to 240 μg/mL) for 9 h, followed by the determination of cellular take frequency using with CLSM.

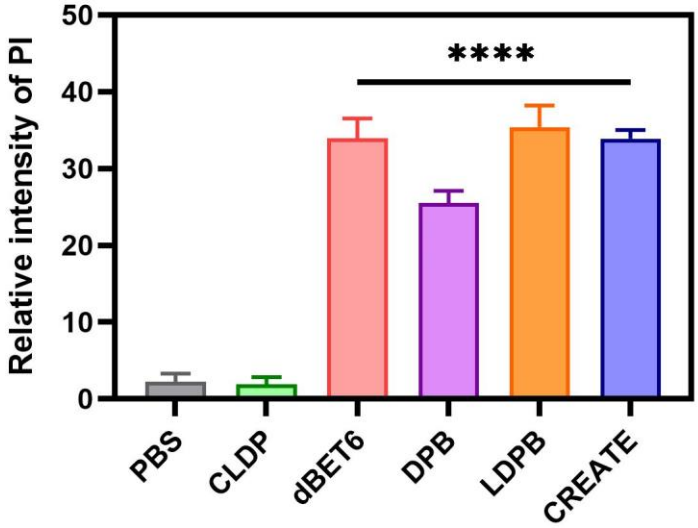

Figure S11. Quantitative analysis of the PI-positive M2-like macrophages. Data are presented as mean ± SD. **** $p < 0.0001$.

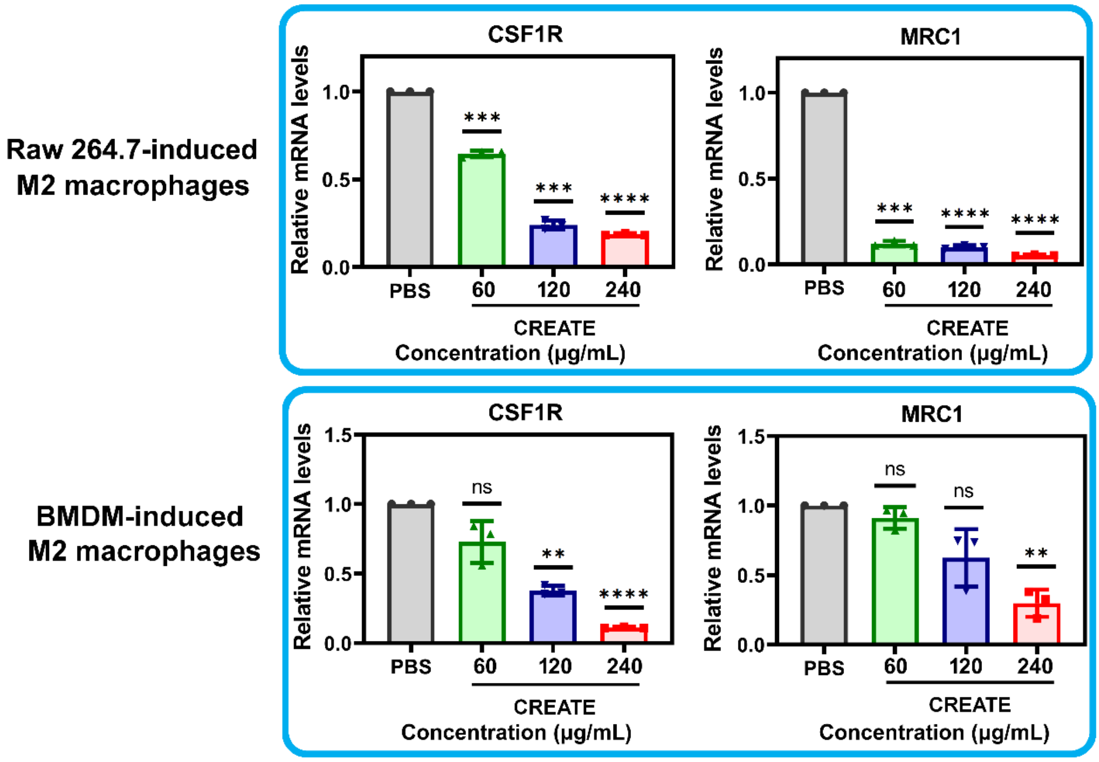

Figure S12. Effect of CREATE on CSF1R and MRC1 mRNA expression in M2-like macrophages. qRT-PCR detection of CSF1R and MRC1 mRNA level in the M2-like macrophages induced by Raw 264.7 and BMDMs, in the presence of different concentrations of CREATE (n=3).

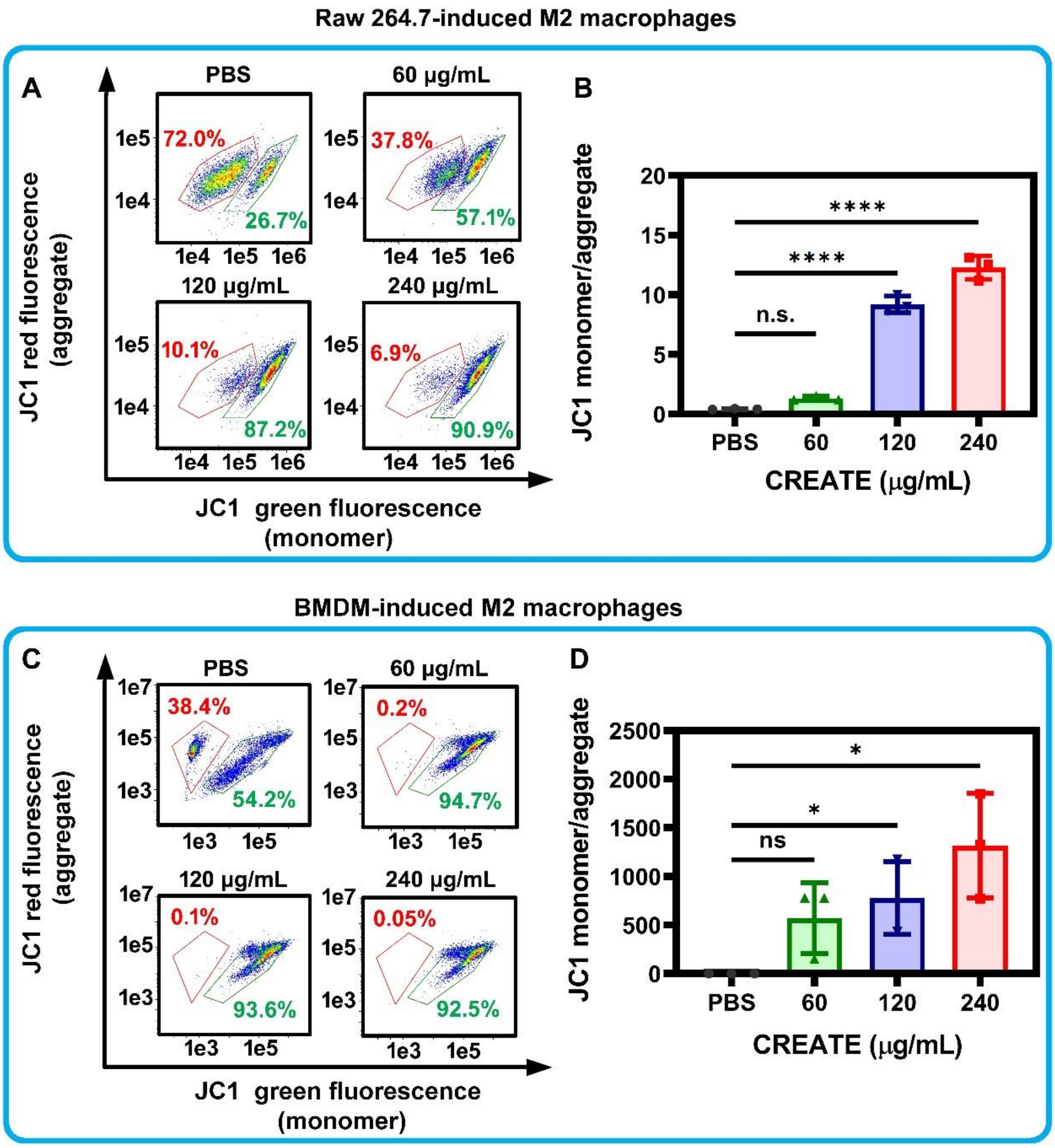

Figure S13. Quantification of JC1 monomers/aggregates of the M2-like macrophages induced from Raw 264.7 (A and B) and BMDMs (C and D). To evaluate the, M2-like macrophages were treated with different concentrations (60 μg/mL, 120 μg/mL, and 240 μg/mL) of CREATE for 9 h, and then incubated with the freshly prepared medium for another 15 h, followed by the analysis of cell viability and apoptosis by flow cytometry (n=3).

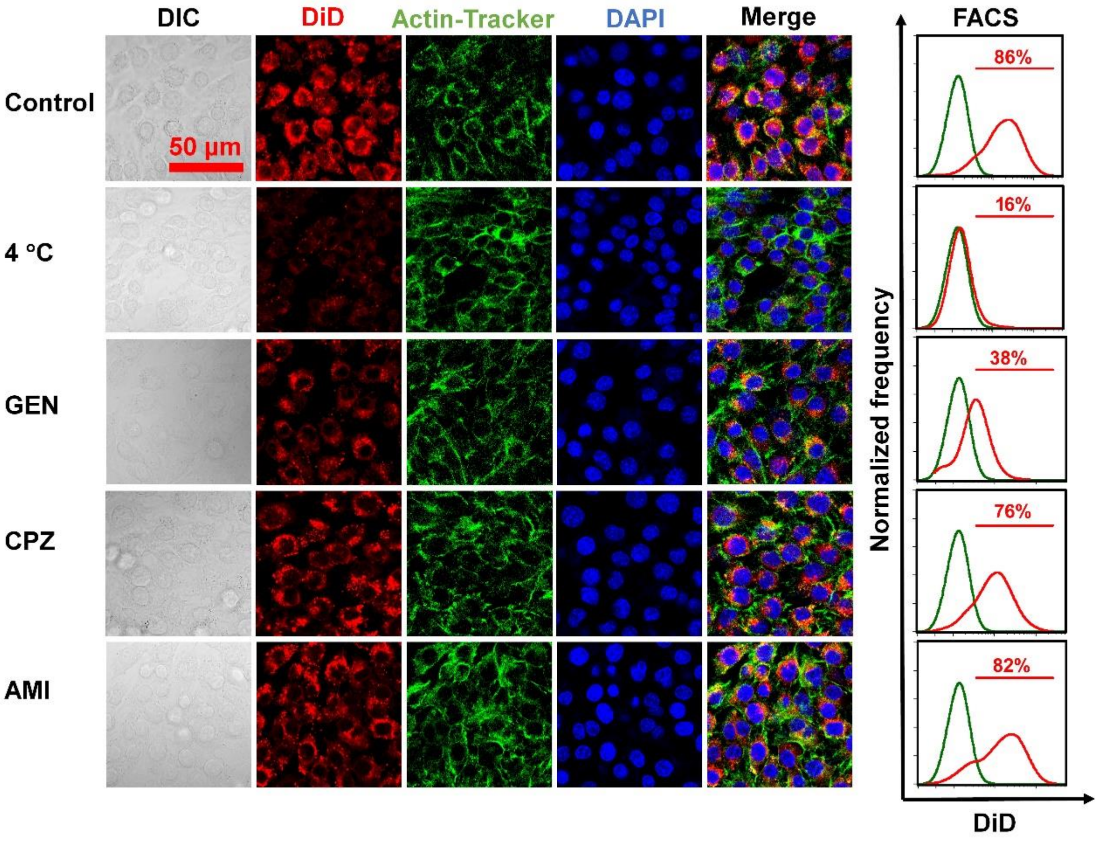

Figure S14. Influence of endocytosis inhibitors on CRV-LLCM/DS-PLGA NPs uptake by LLC cells. Relative uptake efficiency of CRV-LLCM/DS-PLGA/DiD in LLC cells treated with low temperature (4 °C), genistein (GEN), chlorpromazine (CPZ), and Amiloride (AMI), as determined by CLSM and flow cytometry. Data were normalized to cells treated only with CRV-LLCM/DS-PLGA/DiD at 37 °C.

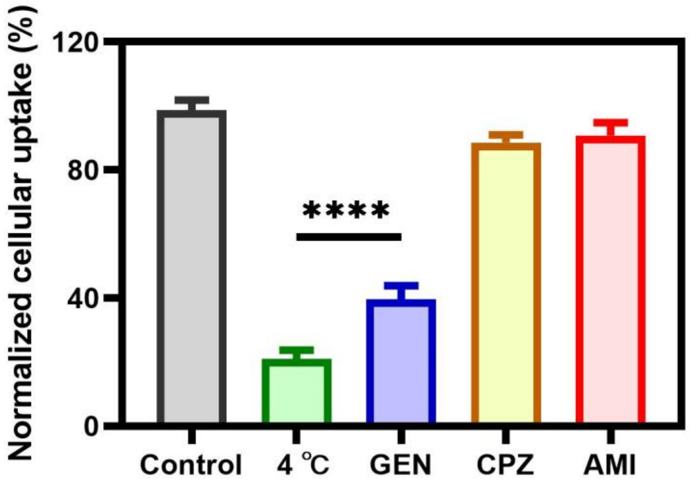

Figure S15. Quantitative analysis of the cellular uptake in LLC cells. Data are presented as mean ± SD. *** $p < 0.001$; **** $p < 0.0001$.

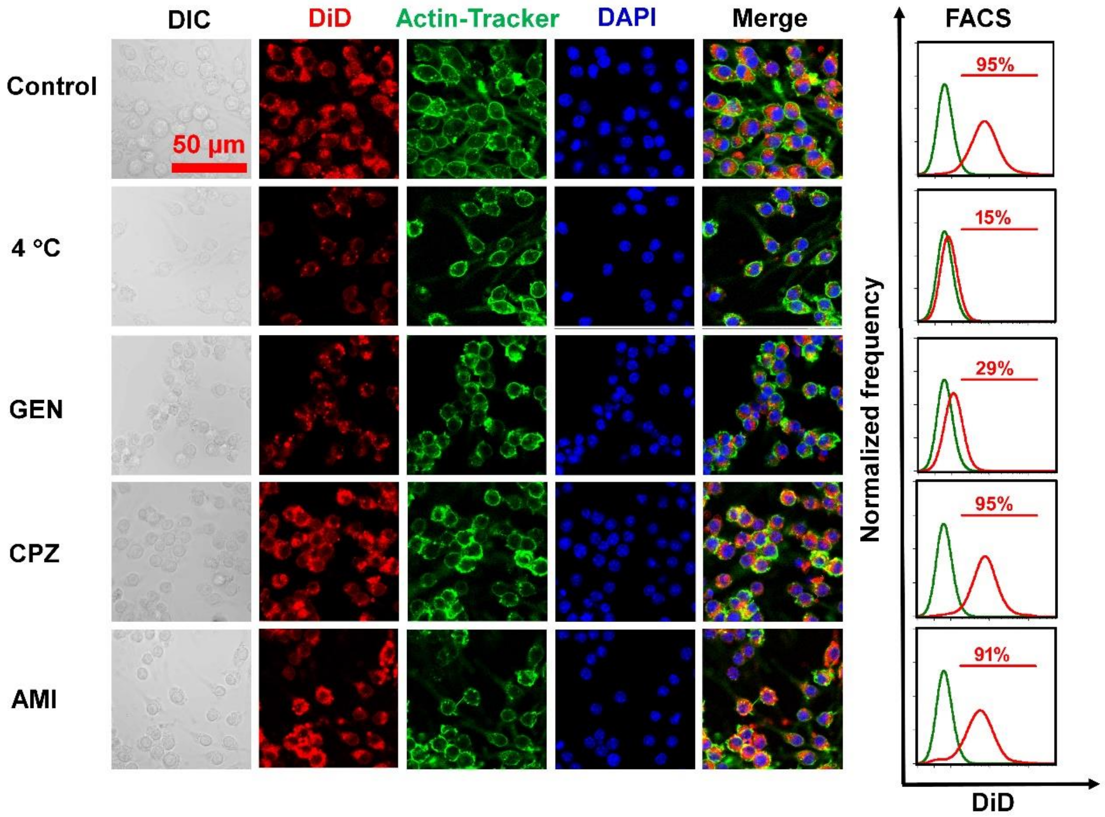

Figure S16. Influence of pharmacological inhibitors on CRV-LLCM/DS-PLGA NPs uptake by M2-like macrophages. Relative uptake efficiency of CRV-LLCM/DS-PLGA/DiD in Raw 264.7-induced M2-like macrophages treated with low temperature (4 °C), genistein (GEN), chlorpromazine (CPZ), and Amiloride (AMI), as determined by confocal and flow cytometry. Data were normalized to cells treated only with CRV-LLCM/DS-PLGA/DiD at 37 °C.

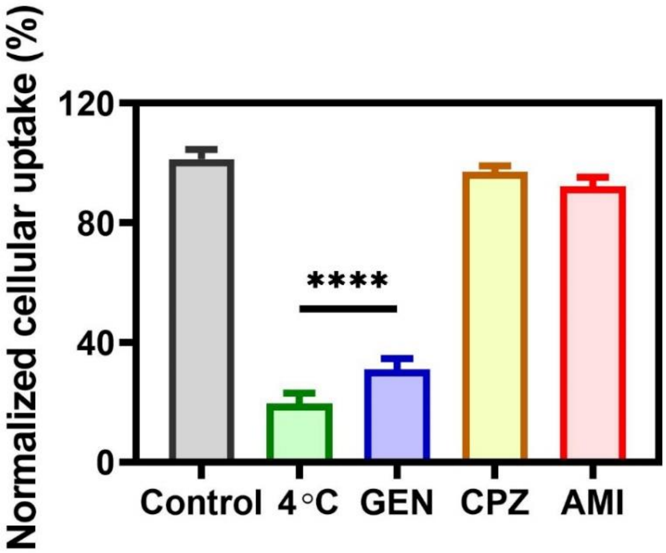

Figure S17. Quantitative analysis of the cellular uptake in M2-like macrophages. Data are presented as mean ± SD. *** $p < 0.001$.

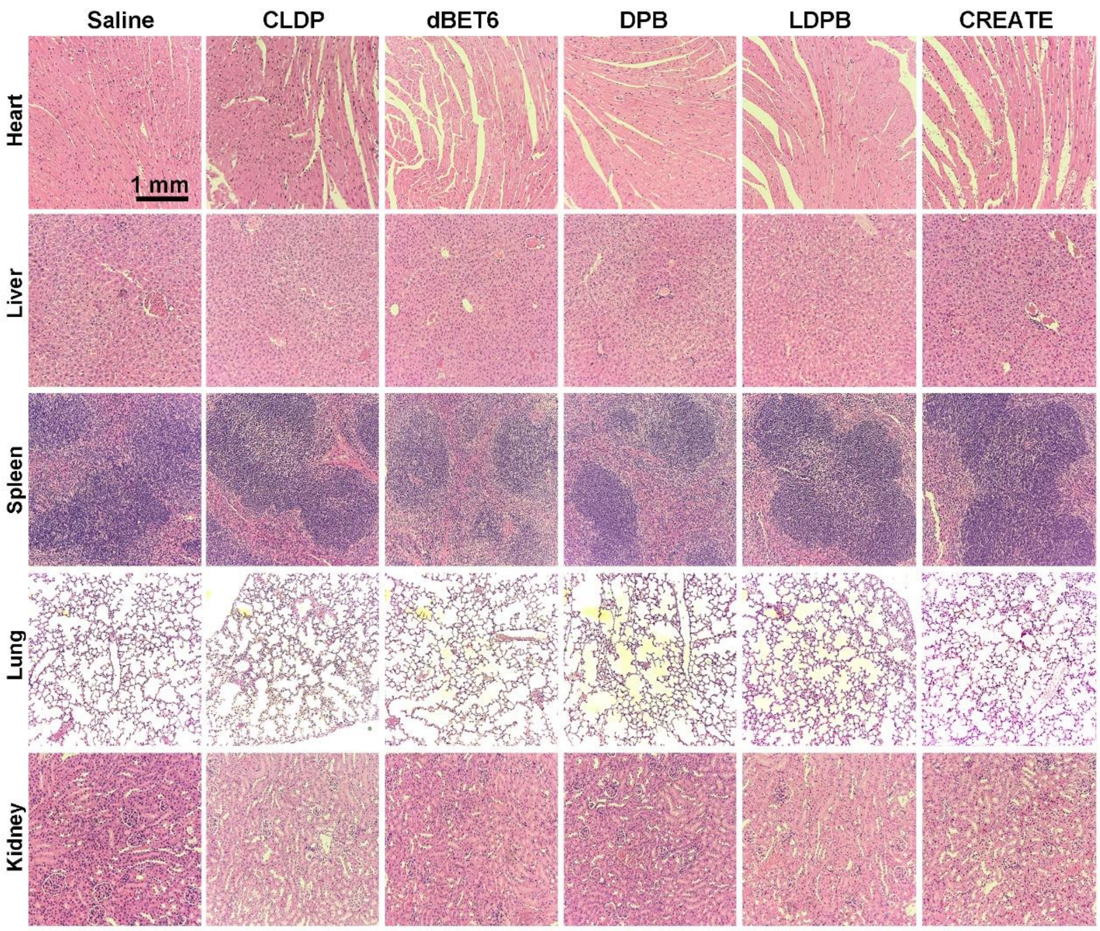

Figure S18. HE staining results of the major organs extracted from LLC tumor-bearing mice.

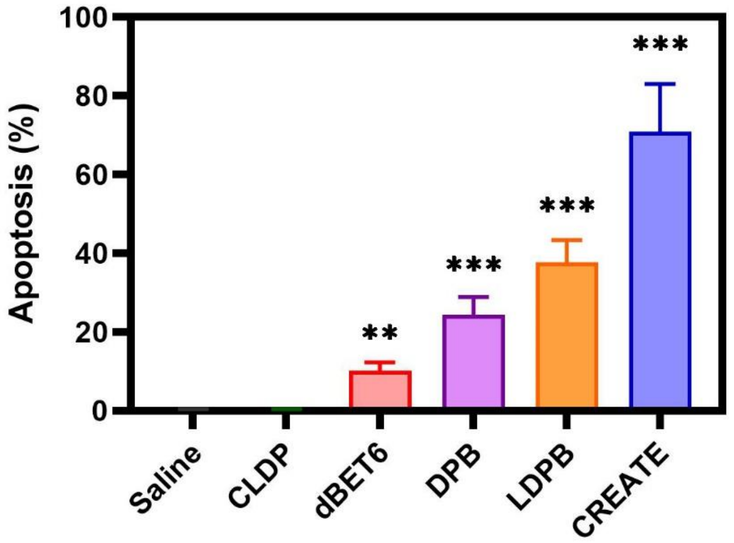

Figure S19. Quantitative analysis of the apoptotic cells. Data are presented as mean ± SEM. ** $p < 0.01$ and *** $p < 0.001$.

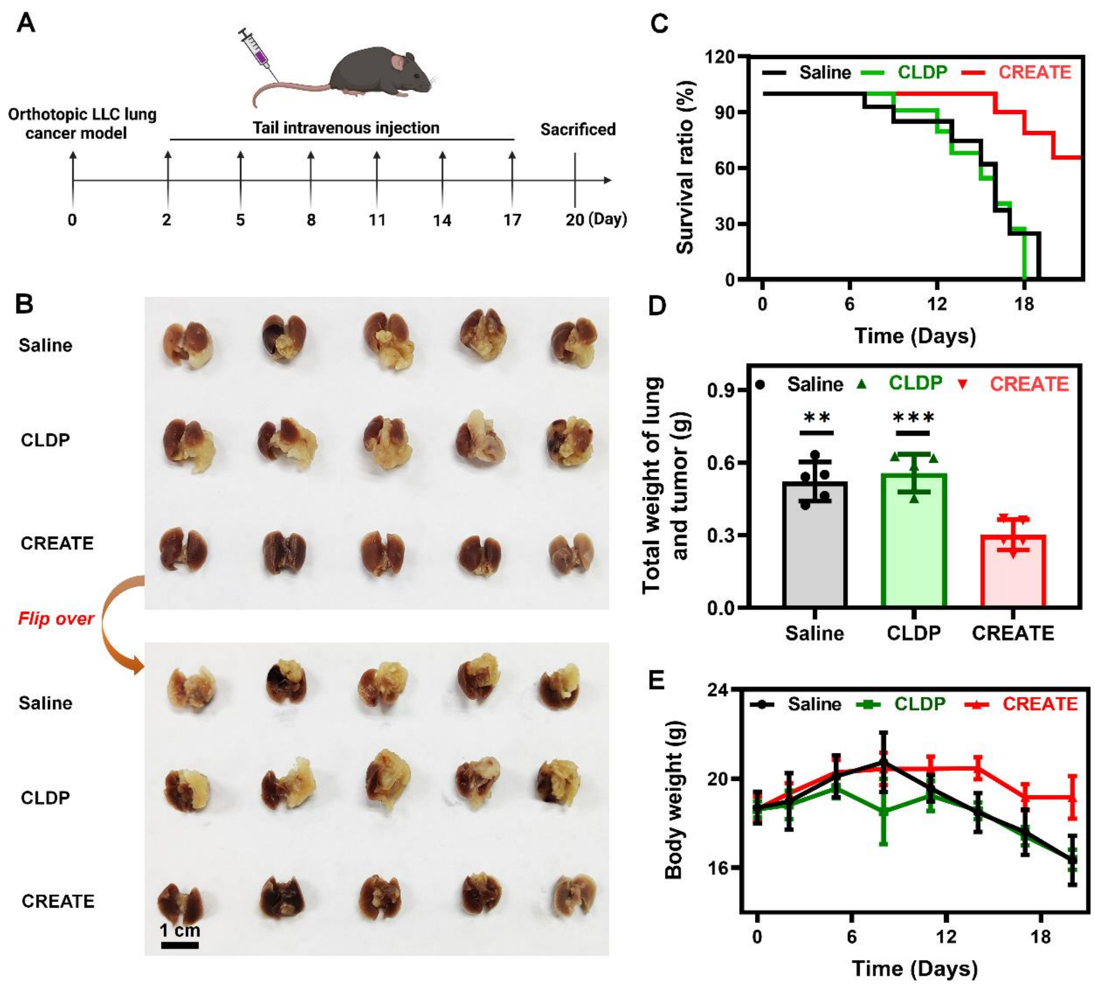

Figure S20. Inhibitory effects of different formulations on orthotopic LLC lung cancer model. (A) Schematic illustrating the *in vivo* therapeutic approach in the orthotopic LLC model. (B) Gross morphology of lung orthotopic tumors after intravenous inoculation with Saline, CLDP, and CREATE extracted from the mice (n=5). (C) Survival curve of mice treated with Saline, CLDP, and CREATE. (D) The total weight of lung and tumor in Saline, CLDP and CREATE groups. (E) The changes of relative body weight detected at the indicated times. Data are presented as mean ± SD. ** $p < 0.01$ and *** $p < 0.001$.

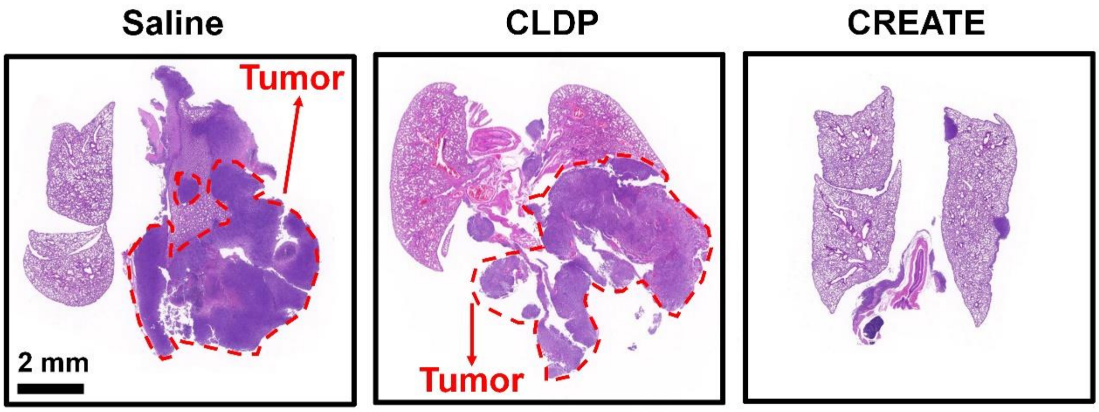

Figure S21. Representative images of HE staining of lung sections treated with Saline, CLDP, and CREATE, respectively. The Dash line indicates the orthotopic tumor.

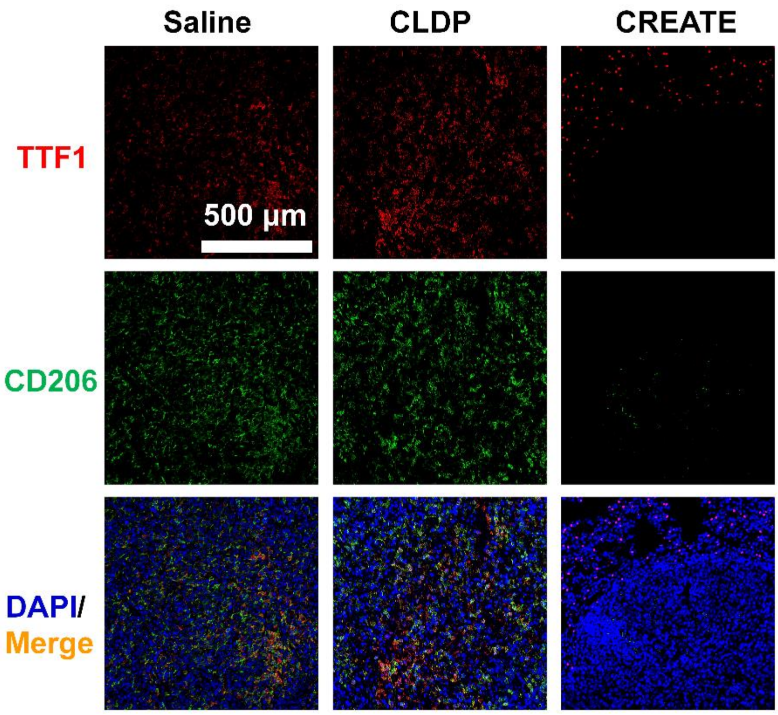

Figure S22. Representative immunofluorescence staining of TTF1^{+} and CD206^{+} cells in the tumor tissues treated with Saline, CLDP, and CREATE.

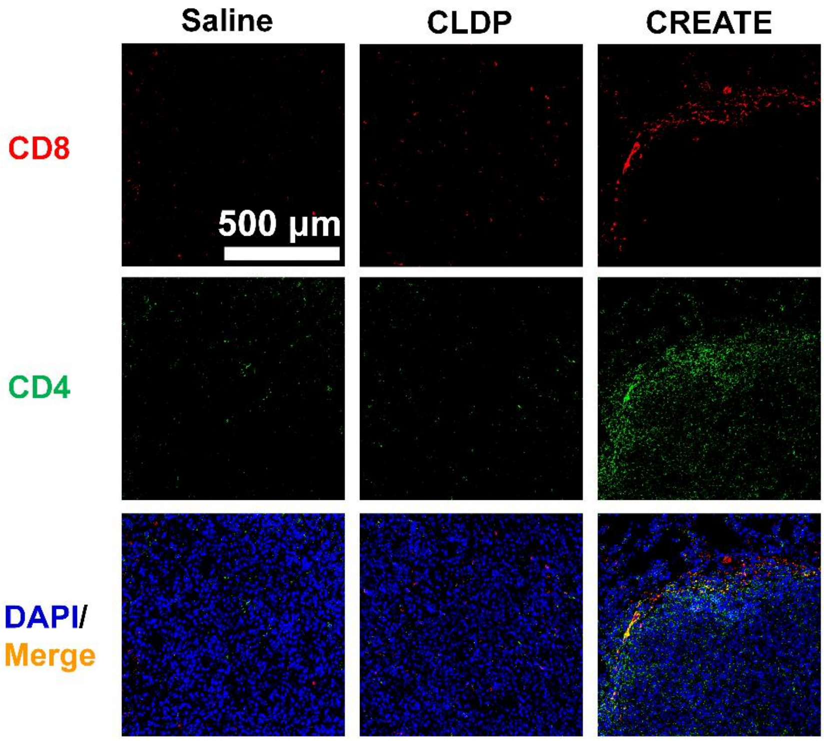

Figure S23. Representative immunofluorescence images of CD4^{+} and CD8^{+} T

cell infiltration in the tumor tissues treated with Saline, CLDP, and CREATE.

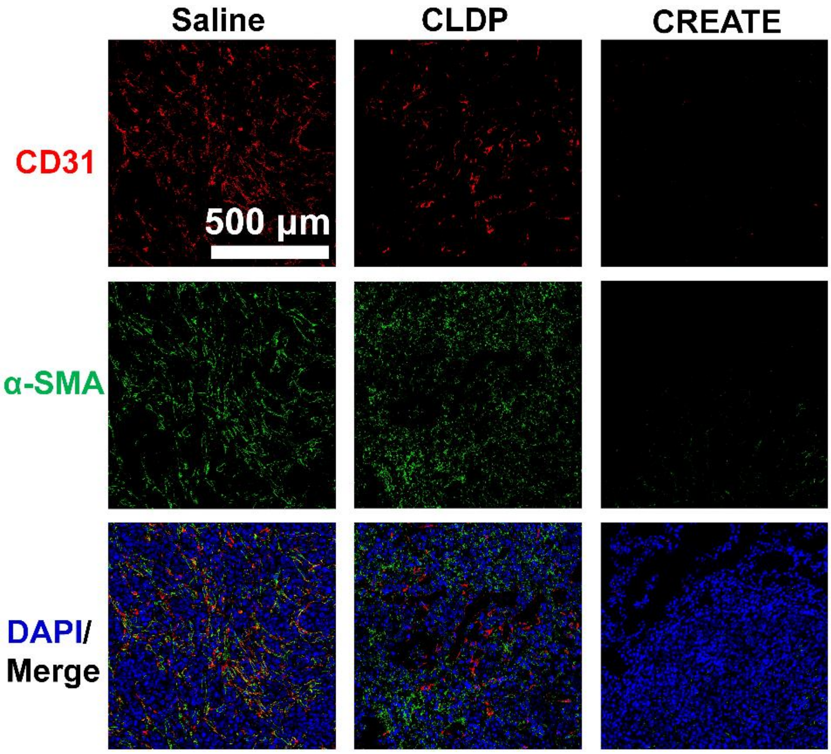

Figure S24. Representative immunofluorescence images of α-SMA^{+} and CD31^{+} tumor blood vessels in the tumor tissues treated Saline, CLDP, and CREATE.
